# SDA: a semi-parametric differential abundance analysis method for metabolomics and proteomics data

**DOI:** 10.1186/s12859-019-3067-z

**Published:** 2019-10-17

**Authors:** Yuntong Li, Teresa W.M. Fan, Andrew N. Lane, Woo-Young Kang, Susanne M. Arnold, Arnold J. Stromberg, Chi Wang, Li Chen

**Affiliations:** 1 Department of Statistics, University of Kentucky, Lexington, 40536 USA; 2 Markey Cancer Center, University of Kentucky, Lexington, 40536 USA; 3 Center for Environmental and Systems Biochemistry, University of Kentucky, Lexington, 40536 USA; 4 Department of Toxicology and Cancer Biology, University of Kentucky, Lexington, 40536 USA; 5 Department of Medicine, University of Kentucky, Lexington, 40536 USA; 6 Department of Biostatistics, University of Kentucky, Lexington, 40536 USA

**Keywords:** Differential abundance analysis, Metabolomics, Proteomics, Semi-parametric log-linear model, Kernel smoothing

## Abstract

**Background:**

Identifying differentially abundant features between different experimental groups is a common goal for many metabolomics and proteomics studies. However, analyzing data from mass spectrometry (MS) is difficult because the data may not be normally distributed and there is often a large fraction of zero values. Although several statistical methods have been proposed, they either require the data normality assumption or are inefficient.

**Results:**

We propose a new semi-parametric differential abundance analysis (SDA) method for metabolomics and proteomics data from MS. The method considers a two-part model, a logistic regression for the zero proportion and a semi-parametric log-linear model for the possibly non-normally distributed non-zero values, to characterize data from each feature. A kernel-smoothed likelihood method is developed to estimate model coefficients and a likelihood ratio test is constructed for differential abundant analysis. The method has been implemented into an R package, *SDAMS*, which is available at https://www.bioconductor.org/packages/release/bioc/html/SDAMS.html.

**Conclusion:**

By introducing the two-part semi-parametric model, SDA is able to handle both non-normally distributed data and large fraction of zero values in a MS dataset. It also allows for adjustment of covariates. Simulations and real data analyses demonstrate that SDA outperforms existing methods.

## Background

Mass spectrometry (MS) has been widely used to profile abundances of metabolomic or proteomic features in biological samples [1]. A common goal of many MS-based studies is to identify features [2, 3] that have different abundances under different experimental groups. For example, in a lung cancer exosomal lipids dataset generated from the Resource Center for Stable Isotope-Resolved Metabolomics at the University of Kentucky, a total of 39 late-stage lung cancer and 27 normal samples were analyzed using Fourier-transform mass spectrometry. The abundances of 282 lipid features were measured. One goal of the study is to identify lipid features that were differentially abundant between lung cancer and normal samples.

**Fig. 1:**
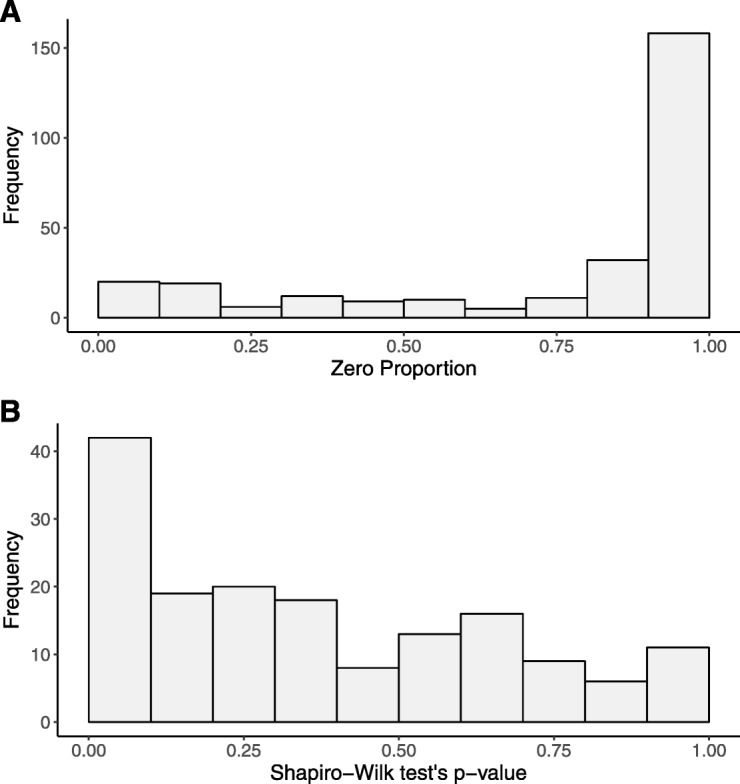
Characteristics of MS data. **a** Distribution of zero value proportions; and **b** Distribution of *p*-values from Shapiro-Wilk tests for features from a lung cancer exosomal lipids dataset. *P*-values were calculated for lung cancer patients and normal controls separately

The MS data sets often contain a large fraction of zero values [4, 5]. For example, in the aforementioned lung cancer exosomal lipid dataset, 40.1% of the observed values were zeros. The distribution of zero value proportion across metabolomic features is presented in Fig. 1a. These zero values indicate the absence or below the detection limit of certain metabolites in certain samples. The existence of these zero values complicates data analysis. Firstly, simply ignoring them would lead to biased results [6, 7]. Secondly, as the data comprise a mixture of a point mass at zero intensity and a distribution of non-zero values, standard statistical methods, such as the two-sample t-test, are inappropriate. To better characterize the data, two-part models, which use one model to quantify the zero proportion and the other model to characterize the non-zero values, have been proposed. Lachenbruch [7] and Taylor and Pullard [8] presented several two-part tests, including the two-part *t*, two-part Wilcoxon and two-part empirical likelihood ratio tests.

Another challenge with the MS data is that the (log-transformed) non-zero values are often non-normally distributed. We applied the Shapiro-Wilk test of normality to each metabolite with at least 20 non-zero values in the lung cancer exosomal lipid dataset. Figure 1b shows the distribution of resulting *p*-values. More than 8% of the *p*-values were less than 0.01, strongly indicating that the abundance data were not normally distributed for at least a substantial number of metabolites. Therefore, differential abundance analysis methods that fit a normal model for the non-zero values of each metabolite, e.g. a two-part t-test [7, 8], are inappropriate and may yield unreliable *p*-values for those non-normally distributed metabolites. As a result, the selection of differentially abundant metabolites is also biased as it is based on the rankings of those suspicious *p*-values that do not compare the significance of different metabolites in a fair and robust manner. Non-parametric methods, such as the two-part Wilcoxon test [7, 8] and empirical likelihood ratio test [8], have also been proposed. However, the tests themselves do not provide a clear quantification of the effect size, do not allow for adjustment of covariates, and may be inefficient.

In this paper, we propose a new semi-parametric differential abundance analysis (SDA) method for proteomics and metabolomics data from mass spectrometry. Our method considers a two-part semi-parametric model to address the issues mentioned above. For the zero part, we consider a logistic regression model which is asymptotically equivalent to the chi-squared test when there is only one categorical experimental factor. For the non-zero part, we consider a semi-parametric log-linear model, which assumes a linear effect of experimental factors on the log-transformed feature abundance but allows an arbitrary distribution for the random error term. The semi-parametric log-linear model has been introduced for survival data, where it is called the semi-parametric accelerated failure time (AFT) model [9]. To our knowledge, this is the first time this model has been utilized for proteomics and metabolomics data, where it is especially attractive because of the ability to handle non-normally distributed data and the direct scientific interpretation of model parameters. In addition, we propose a kernel-smoothed likelihood method to estimate regression coefficients and construct a likelihood ratio test for differential abundant analysis. We evaluate the performance of our method using simulation studies and real data analyses.

## Methods

Our goal is to identify metabolomic or proteomic features that are differentially abundant between experimental groups. As we described in the previous section, MS data comprise a mixture of zero intensity values and possibly non-normally distributed non-zero intensity values. Therefore, the differential abundance analysis needs to be performed to compare both the zero proportion and the mean of non-zero values between groups. To accomplish this, we propose SDA, which considers a two-part semi-parametric model that uses a logistic regression model to characterize the zero proportion and a semi-parametric log-linear model to characterize possibly non-normally distributed non-zero values.

### A two-part semi-parametric model

Let *Y*_*ig*_ be the random variable representing the observed abundance of feature *g* in subject *i* (*i*=1,2,...,*N*). The distribution for *Y*_*ig*_ consists a point mass at zero and a continuous distribution on positive values. We begin by introducing a logistic regression model for the zero part. Let *π*_*ig*_=*Pr*(*Y*_*ig*_=0) be the point mass. We consider

$$\text{log}\left(\frac{\pi_{ig}}{1-\pi_{ig}}\right)=\gamma_{0g}+\boldsymbol{\gamma}_{g} \boldsymbol{X}_{i}, $$

where ***X***_*i*_=(*X*_*i*1_,*X*_*i*2_,...,*X*_*iQ*_)^*T*^ is a *Q*-vector of covariates for subject *i*. The corresponding *Q*-vector of model parameters ***γ***_*g*_=(*γ*_1*g*_,*γ*_2*g*_,...,*γ*_*Qg*_)^*T*^ quantify covariates’ effects on the fraction of zero values for feature *g* and *γ*_0*g*_ is the intercept.

For the continuous non-zero part, i.e. *Y*_*ig*_>0, we consider a semi-parametric model:

$$\text{log}(Y_{ig})=\boldsymbol{\beta}_{g} \boldsymbol{X}_{i} + \varepsilon_{ig}. $$

The model parameters ***β***_*g*_=(*β*_1*g*_,*β*_2*g*_,...,*β*_*Qg*_)^*T*^ have a direct and clear scientific interpretation, i.e. *β*_*qg*_ is the log fold change in observed non-zero abundance comparing different values of the *q*-th covariate for feature *g*. The *ε*_*ig*_’s (*i*=1,2,..*N*) are independent error terms with a common but completely unspecified density function *f*_*g*_. Importantly, we do not impose any distributional assumption on *f*_*g*_. Therefore, our semi-parametric model only specifies a linear effect of covariates, but allows the error term to be arbitrarily distributed. If we further assume *ε*_*ig*_ following a normal distribution, this model reduces to a regular linear regression model on log(*Y*_*ig*_). However, without assuming a specific parametric distribution for *ε*_*ig*_, our model is much more flexible to characterize data with unknown and possibly non-normal distribution.

### Estimation of model parameters

We propose a likelihood-based approach to estimate model parameters. The likelihood function for the two models jointly is:

1 $$ {\begin{aligned} & \prod_{i=1}^{N}\bigg[\frac{\mathrm{exp(\gamma_{0}+\boldsymbol{\gamma}_{g} \boldsymbol{X}_{i})}}{1+\text{exp}(\gamma_{0}+\boldsymbol{\gamma}_{g} \boldsymbol{X}_{i})}\bigg]^{\delta_{ig}}\bigg[\frac{Y_{ig}^{-1}f_{g}(\text{log}(Y_{ig})-\boldsymbol{\beta}_{g} \boldsymbol{X}_{i})}{1+\text{exp}(\gamma_{0}+\boldsymbol{\gamma}_{g} \boldsymbol{X}_{i})} \bigg]^{1-\delta_{ig}},  \end{aligned}}  $$

where *δ*_*ig*_=*I*{*Y*_*ig*_=0} is an indicator function of zero value. Directly calculating the maximum likelihood estimate from this model is intractable because the likelihood involves an infinite-dimensional nuisance parameter *f*_*g*_, which is a common challenge for semi-parametric model inference. A popular approach to overcome this challenge is the nonparametric maximum likelihood (NPML) method [10]. The NPML method restricts the cumulative distribution function of the error term to be a step function and therefore reduces the parameters in the likelihood to finite-dimensional. Then a profile likelihood for the parameters of interest is calculated and the NPML estimate of the parameters of interest is obtained by maximizing the profile likelihood. This approach, however, is infeasible for the semi-parametric model considered here because the resulting profile likelihood depends on the ranks of log(*Y*_*ig*_)−***β***_*g*_***X***_*i*_ and is very non-smooth so that the maximization point of it is unattainable [11].

To address this problem, we replace *ε*_*ig*_’s density function *f*_*g*_(*x*) by its kernel density estimator $1/(Nh)\sum _{j=1}^{n}K\{(\text {log}(Y_{j})-\boldsymbol {\beta }_{g} \boldsymbol {X}_{j} -x)/h\}$, where *K*(·) is a one dimensional kernel function, such as the Gaussian kernel, with bandwidth *h*. Thus, we obtain the following kernel-smoothed approximation of the likelihood in Eq. (1):

$${\begin{aligned} &L(\boldsymbol{\beta}_{g}, \boldsymbol{\gamma}_{g}, \gamma_{0g})\\ &= \prod_{i=1}^{N} \bigg[\frac{\mathrm{exp(\gamma_{0}+\boldsymbol{\gamma}_{g} \boldsymbol{X}_{i})}}{1+\text{exp}(\gamma_{0}+\boldsymbol{\gamma}_{g} \boldsymbol{X}_{i})}\bigg]^{\delta_{ig}} \times\\ &\bigg[\frac{ \frac{1}{Nh} \sum_{j=1}^{N}K\{(\text{log}(Y_{jg})-\boldsymbol{\beta}_{g} \boldsymbol{X}_{j} - (\text{log}(Y_{ig})-\boldsymbol{\beta}_{g} \boldsymbol{X}_{i}))/h\}}{Y_{ig} \{1+\text{exp}(\gamma_{0}+\boldsymbol{\gamma}_{g} \boldsymbol{X}_{i})\}} \bigg]^{1-\delta_{ig}}. \end{aligned}} $$

This kernel-smoothed likelihood includes only a finite number of model parameters. Importantly, this function is very smooth in (*γ*_0*g*_,***γ***_*g*_,***β***_*g*_), and thus the maximum likelihood estimator, ($\hat {\gamma }_{0g}, \hat {\boldsymbol {\gamma }}_{g}, \hat {\boldsymbol {\beta }}_{g}$), can be easily obtained through a trust region maximization algorithm or other Newton-Raphson gradient-based search algorithm [11–14].

### Identification of differentially abundant features

Hypothesis testing on the effect of the *q*-th covariate on the *g*-th feature is performed by assessing *γ*_*qg*_ and *β*_*qg*_. Consider the null hypothesis *H*_0_:*γ*_*qg*_=0 and *β*_*qg*_=0 against alternative hypothesis *H*_1_: at least one of the two parameters is non-zero. We propose a likelihood ratio test (LRT) to test the hypothesis. The test statistic is:

$${LRT_{g} =} -2[\text{log}\{L(\tilde{\gamma}_{0g}, \tilde{\boldsymbol{\gamma}}_{g}, \tilde{\boldsymbol{\beta}}_{g})\}-\text{log}\{L(\hat{\gamma}_{0g}, \hat{\boldsymbol{\gamma}}_{g}, \hat{\boldsymbol{\beta}}_{g}) \}],$$

where ($\tilde {\gamma }_{0g}, \tilde {\boldsymbol {\gamma }}_{g}, \tilde {\boldsymbol {\beta }}_{g}$) is the maximization point of the likelihood under *H*_0_. The *p*-value is calculated based on a chi-square distribution with 2 degrees of freedom. To adjust for multiple comparisons across features, the false discovery rate (FDR) q-value [15] is calculated based on the *qvalue* function in the *qvalue* package in R/Bioconductor.

## Results

### Simulation studies

We performed comprehensive simulation studies to evaluate the performance of SDA and to compare with three existing methods described in Taylor and Pollard [8]: two-part *t* test (2T), two-part Wilcoxon test (2W) and empirical likelihood ratio test (ELRT). Because Taylor and Pollard [8] did not provide a method for multiple comparison adjustment for these three methods, we considered the same FDR adjustment method [15] used in SDA to make methods more comparable.

We focused on the two-group comparison problem and considered two simulation scenarios. For the first scenario, data were simulated based on a prostate cancer proteomics data from the human urinary proteome database [16]. A detailed description of this dataset is provided in the “Real data analyses” section. Each simulated dataset contains 2*n* subjects and 4,000 features. For each feature, the *n* observations of group 1 were generated based on a mixture distribution $p H(x) + (1-p) \hat {F}(x)$, where the zero proportion *p* was generated from Uniform(0,0.8),*H*(*x*) was the unit step function, and $\hat {F}(x)$ was the empirical distribution (in the log scale) of a randomly selected feature that had at least 20 non-zero values in the control group of the proteomics data. For a non-differentially abundant feature, the *n* observations of group 2 were generated from the same distribution as of group 1. For a differentially abundant feature, a 2-fold difference (*β*= log(2)), which was also used in one of the simulation studies in [6], was added to the non-zero part of the distribution.

In our simulations, we set *n* to 50 or 100 and considered 5%, 10% or 20% differentially abundant features. In this section, we only present results from simulations with 10% differentially abundant features. Similar results were obtained for 5% or 20% differentially abundant features (see Additional file 1). For the proposed method, we chose the Gaussian kernel for *K*(·) which is commonly used in kernel density estimation. For the smoothing parameter *h*, we used the optimal bandwidth $h=1.144\hat {\sigma }N^{-1/5}$ [17], where *N*=2*n* is the total sample size, and $\hat {\sigma }$ is the sample standard deviation of {log(*Y*_*ig*_),*i*=1,...*N*}.

**Fig. 2:**
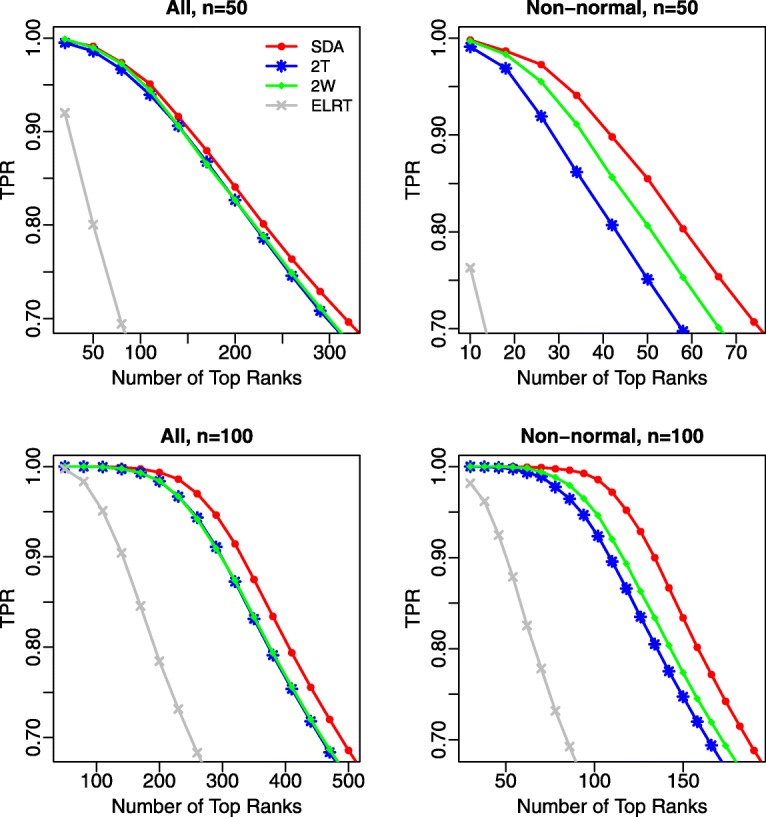
Comparison of the true positive rate (TPR) in top ranked features. Left panels: all features were considered; Right panels: only non-normal features (Shapiro-Wilk test *p*-value <0.01 for at least one of the two groups) were considered. The average TPR over 100 replicates was reported

We first compared the performance of different methods in terms of ranking features. Figure 2 shows the true positive rate (TPR) against the number of top-ranked features based on *p*-values for each method. The left column shows results from all features, including both normally and non-normally distributed. SDA had a higher TPR than all other methods, and the difference increased with sample size. Two-part *t* and two-part Wilcoxon tests had very similar TPRs, while ELRT had a much lower TPR. The right column shows results from non-normally distributed features (Shapiro-Wilk test *p*-value <0.01 for at least one of the two groups). Similar to the left column, SDA had the highest TPR, demonstrating its ability to model non-normally distributed data. The two-part *t*-test had a lower TPR than the two-part Wilcoxon test as the data normality assumption of the two-part *t* test was violated for those features.

To further quantify the overall performance of different methods, we calculated the area under the ROC curve (AUC). As shown in Table 1, SDA had the highest AUC values under all scenarios, especially when evaluating on non-normally distributed features only. The AUCs from two-part Wilcoxon and two-part *t* tests were close to each other when evaluating on all features, and two-part Wilcoxon had a slightly better AUC when evaluating on non-normally distributed features only. ELRT had the worst AUCs in all scenarios.

**Table 1:** Comparison of the area under the ROC curve (AUC)

		All features				Non-normal features			
*n*	DE%	SDA	2T	2W	ELRT	SDA	2T	2W	ELRT
50	5	0.89	0.88	0.88	0.78	0.93	0.88	0.90	0.75
	10	0.89	0.88	0.88	0.78	0.94	0.88	0.91	0.77
	20	0.89	0.88	0.88	0.78	0.93	0.88	0.91	0.76
100	5	0.97	0.95	0.95	0.89	0.98	0.95	0.97	0.88
	10	0.97	0.95	0.95	0.88	0.98	0.95	0.97	0.87
	20	0.97	0.95	0.95	0.89	0.98	0.95	0.96	0.88

The AUCs based on all features and non-normal features (Shapiro-Wilk test *p*-value <0.01 for at least one of the two groups) were both reported. Results were based on an average over 100 replicates

**Fig. 3:**
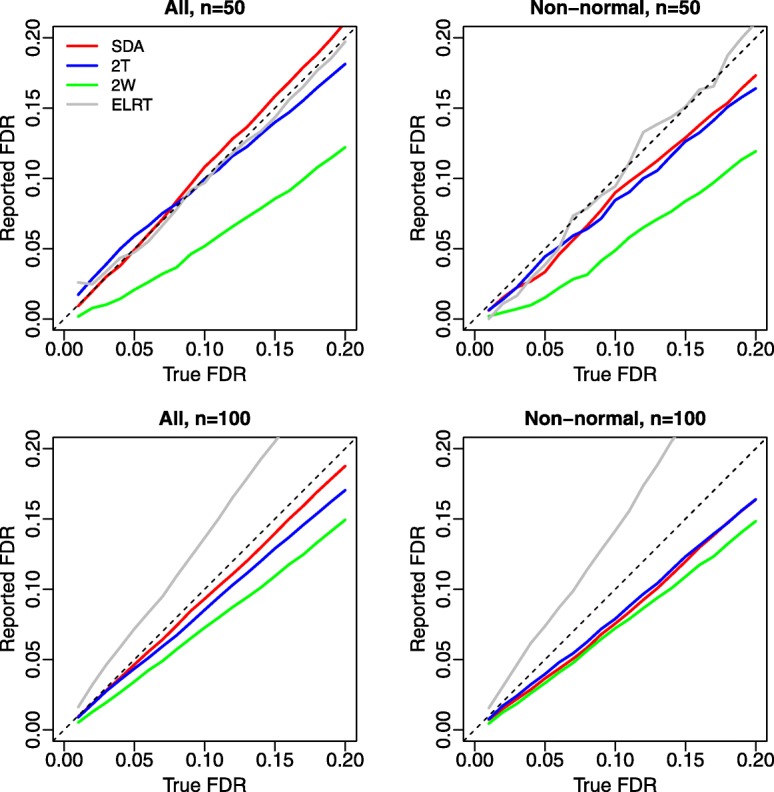
Comparison of false discovery rate (FDR) estimation. Left panels: all features were considered; Right panels: only non-normal features (Shapiro-Wilk test *p*-value <0.01 for at least one of the two groups) were considered. Results were averaged over 100 replicates

We next assess the accuracy in estimating the FDR for different methods. Figure 3 displays the reported FDR against true FDR. The reported FDR based on SDA and two-part *t*-test were close to the true FDR, indicating that those methods were able to accurately estimate the FDR. The reported FDR based on the two-part Wilcoxon test was smaller than the true FDR under all scenarios, suggesting that it was conservative in detecting differentially abundant features. The reported FDR based on ELRT was close to the true FDR when *n*=50, but went larger than the true FDR when *n* increased to 100.

**Fig. 4:**
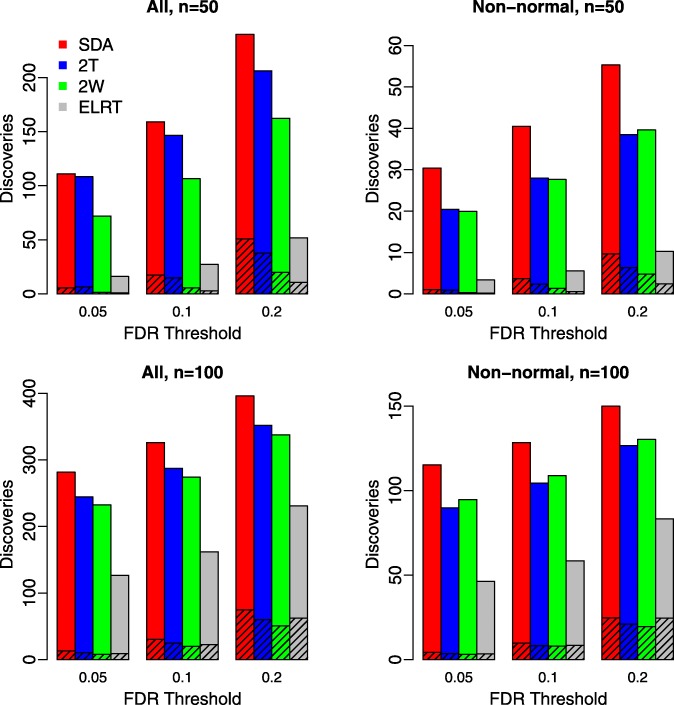
Comparison of the number of significant features for an FDR threshold of 0.05, 0.1, or 0.2. The unshaded bar indicates the number of true discoveries, and the shaded bar indicates the number of false discoveries. Results were averaged over 100 replicates. Left panels: all features were considered; Right panels: only non-normal features (Shapiro-Wilk test *p*-value <0.01 for at least one of the two groups) were considered

Figure 4 plots the number of discoveries against a given FDR threshold, which was set to 0.05, 0.1, or, 0.2. For each scenario, we present the total discoveries as well as the false discoveries (shaded area). The SDA method identified more truly differentially abundant features than all other methods at any given threshold.

For the second simulation scenario, data were simulated following the same procedure as the first simulation scenario, but with one additional step of censoring by a detection limit. Specifically, the detection limit for a feature was chosen as the 10th percentile of the simulated non-zero values from the two groups combined. All non-zero values below the detection limit were set to zero to mimic the situation that a fraction of observed zero values were due to detection limit. Data simulated under this scenario had different numbers of zeros between groups for differentially abundant features because the group with lower abundance level of a feature had more values that fell below the detection limit. The results were presented in Figures S7-15 in Additional file 1. Similar to the first simulation scenario, SDA had a higher true positive rate compared to other methods under this simulation scenario. SDA also identified more truly differentially abundant features than all other methods at any given FDR threshold for non-normally distributed features.

### Real data analyses

#### Prostate cancer proteomics data

**Fig. 5:**
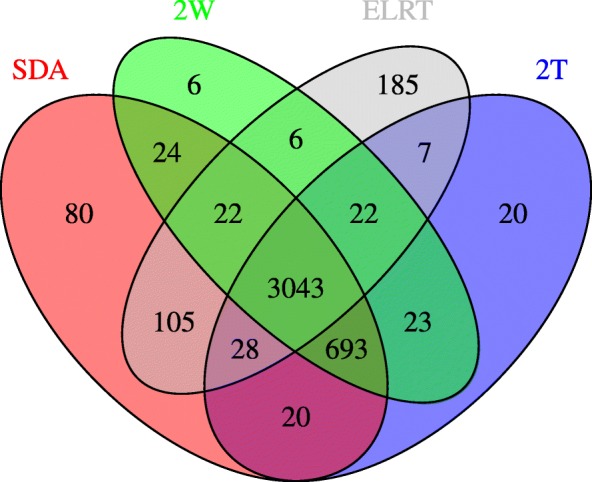
A Venn diagram visualizing the number of distinct and common differentially abundant features identified by each method based on the prostate cancer proteomics data. The FDR threshold was 0.05

**Fig. 6:**
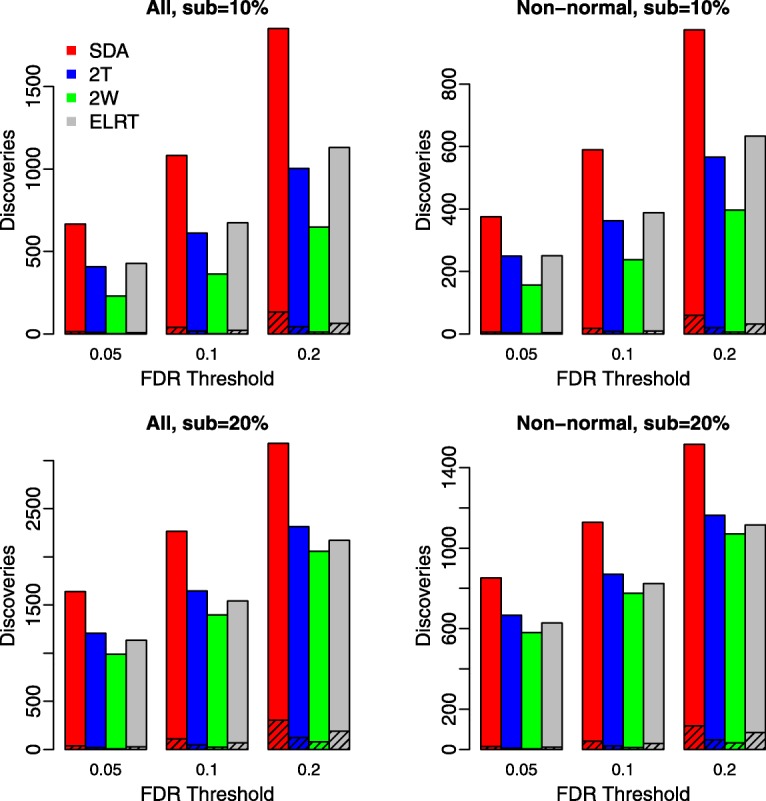
Concordance between the sub- and whole dataset differential abundance analysis based on the prostate cancer proteomic data. The FDR threshold was 0.05. The unshaded bar indicates the number of differentially abundant features from the sub-dataset analysis which were also identified by the whole dataset analysis, and the shaded bar indicates the number of differentially abundant features from the sub-dataset analysis which were not identified by the whole dataset analysis. Results were averaged over 100 replicates. Upper panels: sub-sampling 10% of the data; lower pannels: sub-sampling 20% of the data. Left panels: all features were considered; Right panels: only non-normal features (Shapiro-Wilk test *p*-value <0.01 for at least one of the two groups) were considered

We applied our method to prostate cancer data from the human urinary proteome database [16]. In our analysis, we compared proteomic feature abundances between 526 prostate cancer and 1503 healthy subjects. A total of 5605 proteomic features were measured for each subject, where the abundance measurement had been normalized relative to 29 urinary “housekeeping” peptides to adjust for analytical and urine dilution variances [16, 18, 19]. Figure 5 presents results on analyzing the whole dataset with an FDR threshold of 0.05. The majority of differentially abundant features identified by different methods overlapped, having 3043 features in common. We next evaluated the performance of different methods under smaller sample size, where we sub-sampled 10% or 20% of the data and calculated the concordance on identified differentially abundant features between the sub- and whole datasets. Specifically, we focused on the 3043 features that were commonly identified by all methods from the whole dataset and investigated what fraction of these features could also be identified by each method when analyzing the sub-dataset. Figure 6 plots the number of discoveries under FDR threshold of 0.05, 0.1 or 0.2. Compared to other methods, SDA based on a sub-dataset were able to identified a larger number of the 3043 differentially abundant features obtained from the whole dataset, and therefore provided a better concordance between the sub- and whole dataset analysis.

#### Lung cancer exosomal lipids data

We applied our method to the lung cancer exosomal lipids dataset described in the “Background” section. The data acquisition and normalization procedure of this dataset is provided in Additional file 2. Table 2 shows differentially abundant features identified by SDA, two-part *t*, two-part Wilcoxon, and ELRT tests for the comparison between late stage lung cancer and normal samples. SDA identified a total of 15 differentially abundant features, including all 6 features identified by any of the other three methods and 9 additional features. These features were further characterized by tandem MS, which showed that several ions comprise more than one isobaric species which could be assigned to specific lipids (see Table S1 in Additional file 2). The lipids were dominated by triglycerides, which are typically storage lipids and associated with lung cancer risk based on cohort studies [20, 21]. Some of the acyl chains were long chain (>16) and polyunsaturated, which can be hydrolyzed to bioactive lipids (diacylglycerols and the fatty acids). Also found was a sphingomyelin, which can be important cell signaling regulators [22] with key roles in lung cancer pathogenesis [23].

**Table 2:** Differentially abundant features identified by different methods based on the lung cancer exosomal lipids data

Feature ID	$\hat {\gamma }_{g}$	$\hat {\beta }_{g}$	*q*_*SDA*_	*q*_2 *T*_	*q*_2 *W*_	*q*_*ELRT*_
C47H86O6	0.56	-1.17	0.02	0.01	0.25	—
C53H94O6	1.97	-0.7	0.02	0.02	0.08	—
C57H108O6*	1.13	-0.89	0.02	0.18	0.3	0.33
C59H104O6	2.54	-0.23	0.02	0.03	0.08	—
C54H100O6	1.3	-0.57	0.04	0.07	0.14	—
C49H92O6*	1.3	-0.66	0.05	0.26	0.32	0.33
C39H79N2O6P1*	—	0.38	0.07	0.7	0.74	0.73
C40H80N1O8P1*	—	0.31	0.07	0.38	0.32	0.33
C51H94O6*	1.87	-0.48	0.07	0.26	0.32	0.33
C52H98O6*	0.59	-0.8	0.07	0.18	0.32	—
C56H104O6*	0.99	-0.57	0.07	0.13	0.25	—
C56H106O6	-0.3	-0.94	0.07	0.04	0.3	—
C59H106O6*	1.03	-0.7	0.07	0.17	0.25	—
C59H112O6	-0.49	-0.91	0.07	0.01	0.13	—
C56H102O6*	1.13	-0.54	0.08	0.18	0.3	—

FDR threshold was 0.1. Estimations of *γ* and *β* as well as q-values from different methods are presented. Lipid assignments of those features are provided in Table S1 in Additional file 2. * indicates features only identified by SDA. — indicates results not available. For C39H79N2O6P1 and C40H80N1O8P1, the calculation of $\hat {\gamma }_{g}$ is not available because there is no zero value in the cancer samples. For the ELRT method, q-values for many features were not available

## Discussion

In standard statistical practice, examining data normality is usually the first step of data analysis. If the data is normally distributed, parametric methods, e.g. t-test, will be used. Otherwise, non-parametric tests, e.g. Wilcoxon rank-sum test, will be considered. However, for metabolomics and proteomics data with a large number of features, it is more difficult to examine data normality for each of the features, but the choice of an appropriate statistical method depends on it. SDA solves this problem by introducing a unified semi-parametric model for both normally and non-normally distributed data, and therefore providing valid inference without having to check for data normality. SDA possesses merits of both non-parametric and parametric methods. On one hand, it is free of the data normality assumption. On the other hand, it allows quantification of the effect size and adjustment of covariates.

SDA is robust to the choice of bandwidth for moderate to large sample size. But when the sample size is small, choice of bandwidth may have an impact. We evaluated the bandwidth proposed by [17] as well as five other bandwidths described in [11] using simulation studies. We found that the bandwidth $h=1.144\hat {\sigma }N^{-1/5}$ [17] yielded the best performance (data not shown). Therefore, this bandwidth was used in our analysis.

The observed zero values may be a mixture of zeros due to the absence of a compound and values below the detection limit. To deal with values below the detection limit, one frequently used approach is data imputation [24]. However, for MS data, it is unknown whether an observed zero value is due to the absence of a compound or below the detection limit. Data imputation can only be performed on all the observed zero values, which would lead to biased results because zero values due to the absence of a compound would also be imputed with positive values. In fact, it is difficult to distinguish these two types of zeros in statistical inference without imposing additional parametric model assumptions. Therefore, our method, as well as the two-part t-test and two-part Wilcoxon test, focuses on assessing the null hypothesis that the distribution of observed abundance level is the same between groups, i.e. the proportion of observed zero values (including both the absence of a compound and below the detection limit) and the distribution of observed non-zero values (values above the detection limit) are the same between groups. Our alternative hypothesis is that the proportion of observed zero values and/or the distribution of observed non-zero values are different between groups.

For the case of two-group comparison in presence of detection limit, our test is also a valid test (in terms of preserving the type I error rate) for assessing the null hypothesis that the distribution of underlying abundance level without censoring by the detection limit is the same between groups, i.e. the proportion of zero underlying abundance values and the distribution of non-zero underlying abundance values are the same between groups (see Proposition S1 in Additional file 3). To numerically validate this, we performed a single-feature simulation study, which showed that our test preserved the type I error rate around 5% (see Table S2 in Additional file 1). Furthermore, as demonstrated by the second simulation scenario in the “Simulation studies” section, our method outperformed other methods in identifying differentially abundant features, especially non-normally distributed features, under such situation.

This paper focuses on downstream differential abundance analysis of MS data, expecting that the data have already been appropriately processed and normalized. In fact, data normalization is a critical step in MS data processing to adjust size effect, due to the difference in the sample amount or dilution across samples, as well as other technical variations. Various data normalization methods, such as housekeeping normalization [18, 25, 26], centred logratio transformation [25], probabilistic quotient normalization [25, 27], total sum normalization [25], and variance stabilization normalization [27, 28], have been proposed. The choice of an appropriate normalization method depends on the type of biological samples, the study design, and the investigator’s experience. It has been shown that data normalization can substantially affect downstream analysis [25, 28, 29]. Therefore, we highly suggest users to carefully perform data normalization prior to differential abundance analysis.

We consider the case that individual observations are independent of each other in this paper. One of our future directions is to extend SDA to paired data, e.g. comparing metabolomic profiles between paired tumor and normal samples from the same patient. To deal with the correlation between paired samples, we can introduce random effect terms in both the logistic regression and the semi-parametric log-linear models. However, the computation of kernel-smoothed likelihood is more complicated.

## Conclusion

In this paper, we propose a new differential abundance analysis method, SDA, for metabolomic and proteomic data generated from MS. Based on a two-part semi-parametric model, the SDA method is able to robustly handle non-normally distributed data and to adjust for covariates. Meanwhile, our model provides a direct quantification of the effect size. We develop a kernel-smoothed likelihood procedure for model parameter estimation and a likelihood ratio test for differential abundance analysis. Simulation studies and analyses of proteomics and metabolomics datasets show that SDA outperforms existing methods.

## Supplementary information

**Additional file 1** Additional simulation results. This file provided additional simulation results with 5% or 20% differentially abundant features for the first simulation scenario described in the main text. We compared SDA to 2T, 2W and ELRT methods for true positive rate, FDR and number of significant features with a given FDR threshold. SDA performed better than other methods in all comparisons. This file also provided simulation results with 5%, 10% or 20% differentially abundant features for the second simulation scenario described in the main text. SDA also outperformed other methods in identifying differentially abundant features, especially non-normally distributed features. In addition, this file provided results from a single-feature simulation study showing that SDA preserved the type I error rate around 5% for two-group comparison in presence of detection limit.

**Additional file 2** Data acquisition procedure, data normalization and lipid assignment of differentially abundant features for the lung cancer exosomal lipid dataset.

**Additional file 3** A proposition to show that for the case of two-group comparison in presence of detection limit, our test is also a valid test (in terms of preserving the type i error rate) to assess the null hypothesis that the distribution of underlying abundance level without censoring by the detection limit is the same between groups.

## Data Availability

An R package, *SDAMS*, that implements the proposed method is available at https://www.bioconductor.org/packages/release/bioc/html/SDAMS.html. The code for performing simulation studies and reproducing figures/tables is available at http://sweb.uky.edu/~cwa236/SDA.html.

## Abbreviations

2T: two-part *t* test
2W: two-part Wilcoxon test
AFT: Accelerated failure time
AUC: Area under the ROC curve
ELRT: Empirical likelihood ratio test
FDR: False discovery rate
LRT: Likelihood ratio test
MS: Mass spectrometry
NPML: Nonparametric maximum likelihood
ROC: Receiver operator characteristic
SDA: Semi-parametric differential abundance analysis
TPR: True positive rate
