# Supplementary material for: SDA: a semi-parametric differential abundance analysis method for metabolomics and proteomics data
Source: BMC Bioinformatics. 2019 Oct 17;20:501. doi: 10.1186/s12859-019-3067-z (PMC6798423; doi:10.1186/s12859-019-3067-z)
Supplement: Supplementary file 1 — Additional file 1 Additional simulation results. This file provided additional simulation results with 5% or 20% differentially abundant features for the first simulation scenario described in the main text. We compared SDA to 2T, 2W and ELRT methods for true positive rate, FDR and number of significant features with a given FDR threshold. SDA performed better than other methods in all comparisons. This file also provided simulation results with 5%, 10% or 20% differentially abundant features for the second simulation scenario described in the main text. SDA also outperformed other methods in identifying differentially abundant features, especially non-normally distributed features. In addition, this file provided results from a single-feature simulation study showing that SDA preserved the type I error rate around 5% for two-group comparison in presence of detection limit. [file 12859_2019_3067_MOESM1_ESM.pdf]

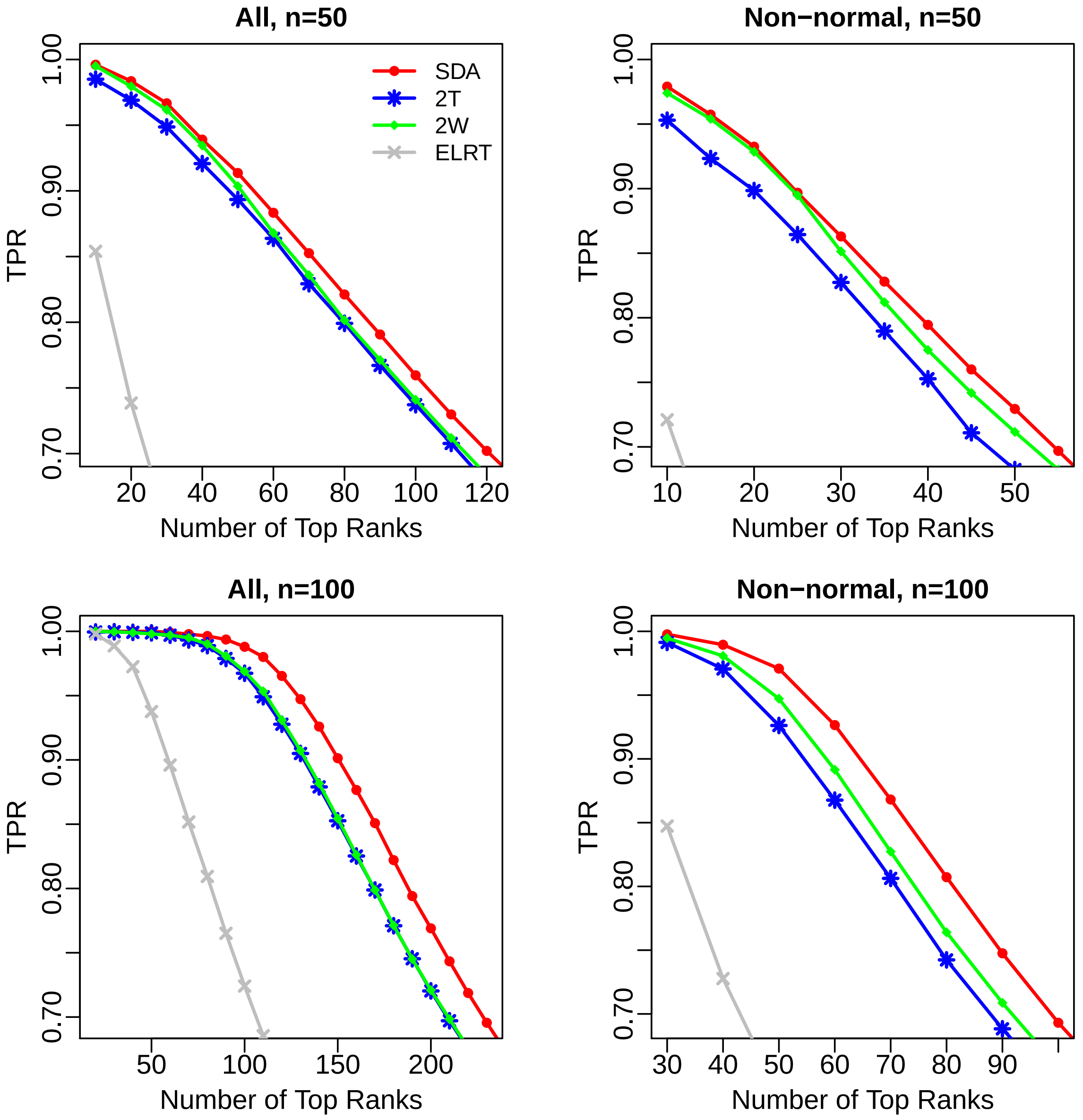

Figure S1: Comparison of the true positive rate (TPR) in top ranked features when 5% of features were truly differentially abundant based on the first simulation scenario. Left panels: all features were considered; Right panels: only non-normal features (Shapiro-Wilk test p-value < 0.01 for at least one of the two groups) were considered. The average TPR over 100 replicates was reported.

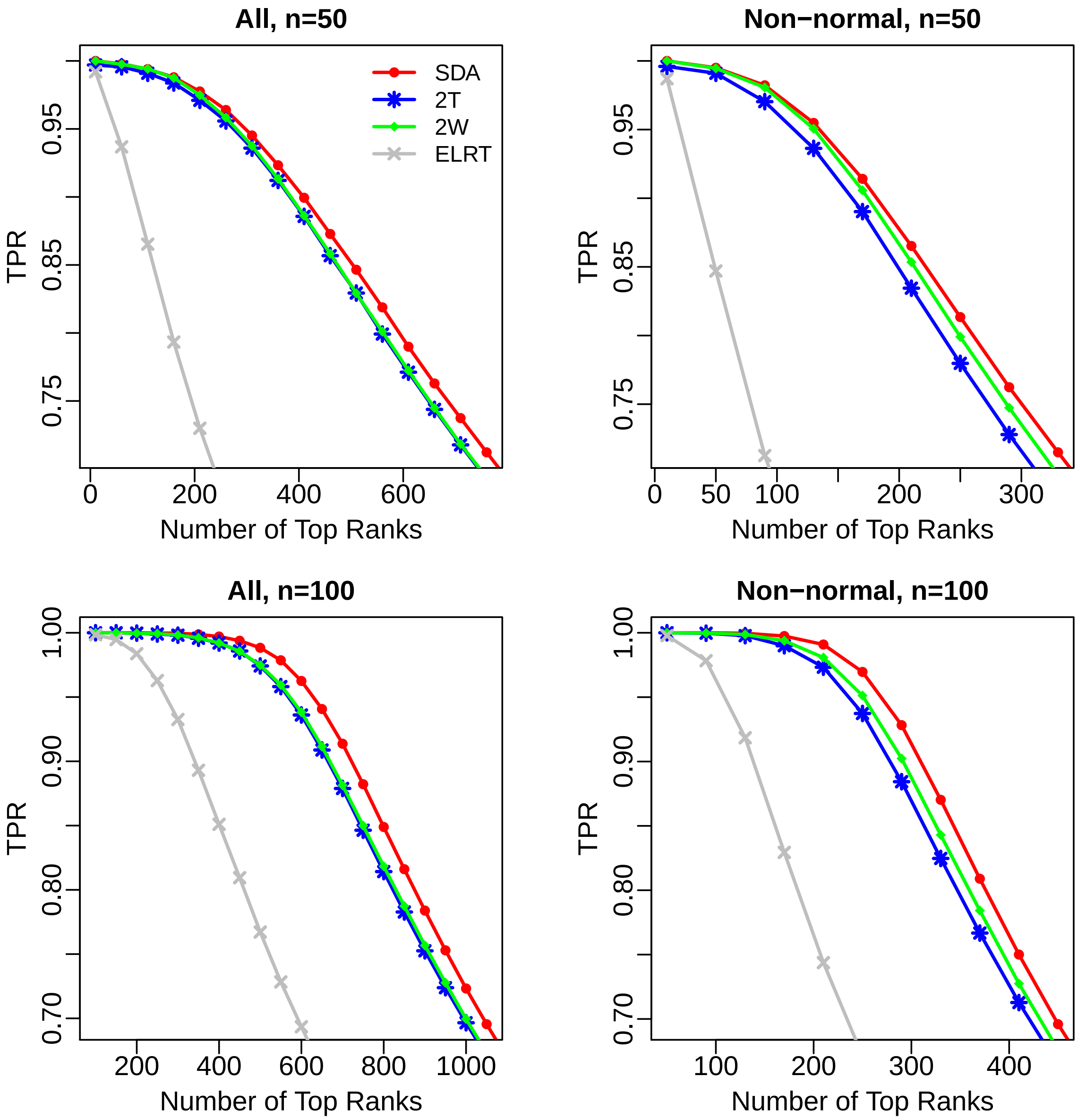

Figure S2: Comparison of the true positive rate (TPR) in top ranked features when 20% of features were truly differentially abundant based on the first simulation scenario. Left panels: all features were considered; Right panels: only non-normal features (Shapiro-Wilk test p-value < 0.01 for at least one of the two groups) were considered. The average TPR over 100 replicates was reported.

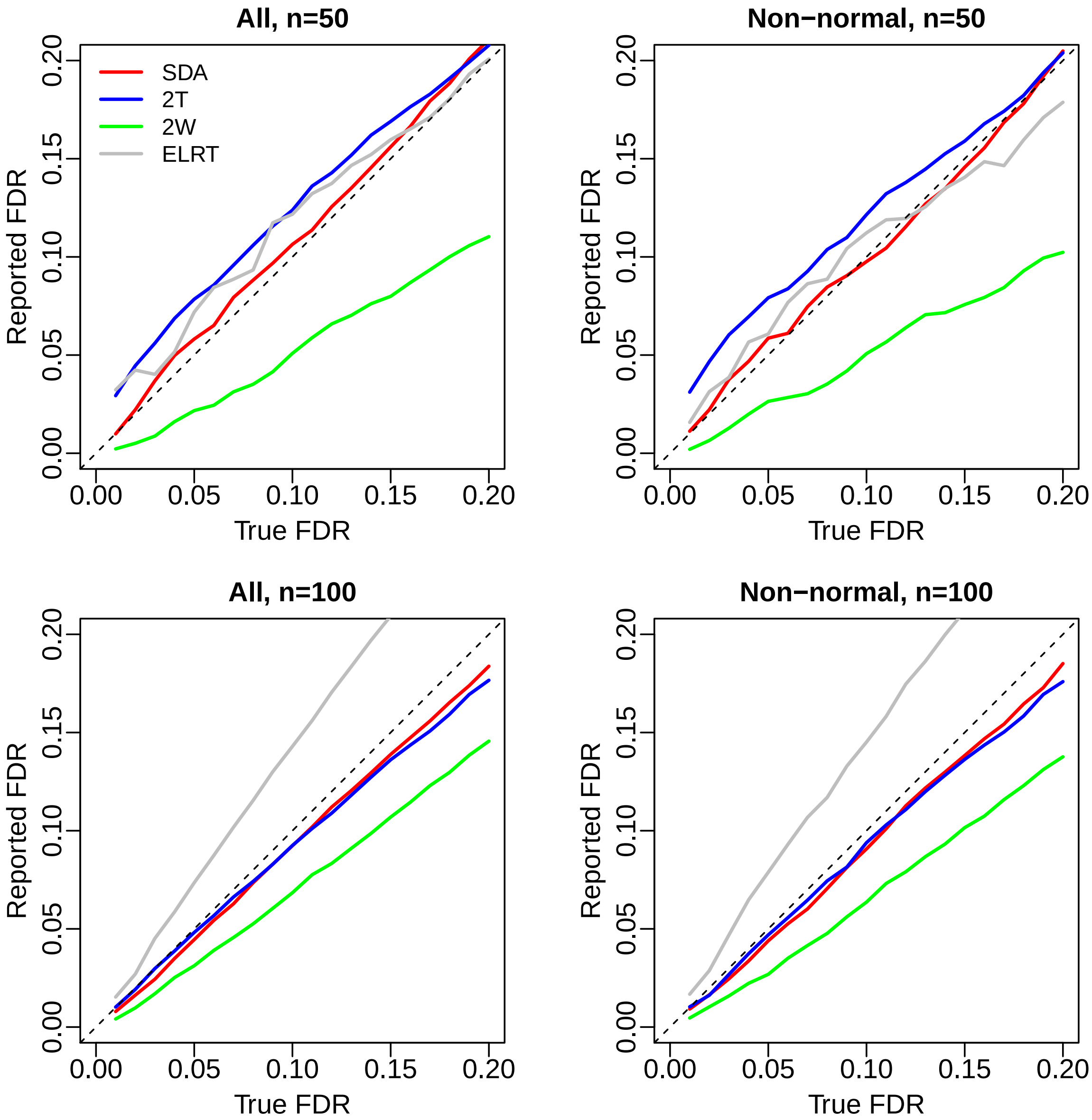

Figure S3: Comparison of false discovery rate (FDR) estimation when 5% of features were truly differentially abundant based on the first simulation scenario. Left panels: all features were considered; Right panels: only non-normal features (Shapiro-Wilk test p-value < 0.01 for at least one of the two groups) were considered. Results were averaged over 100 replicates.

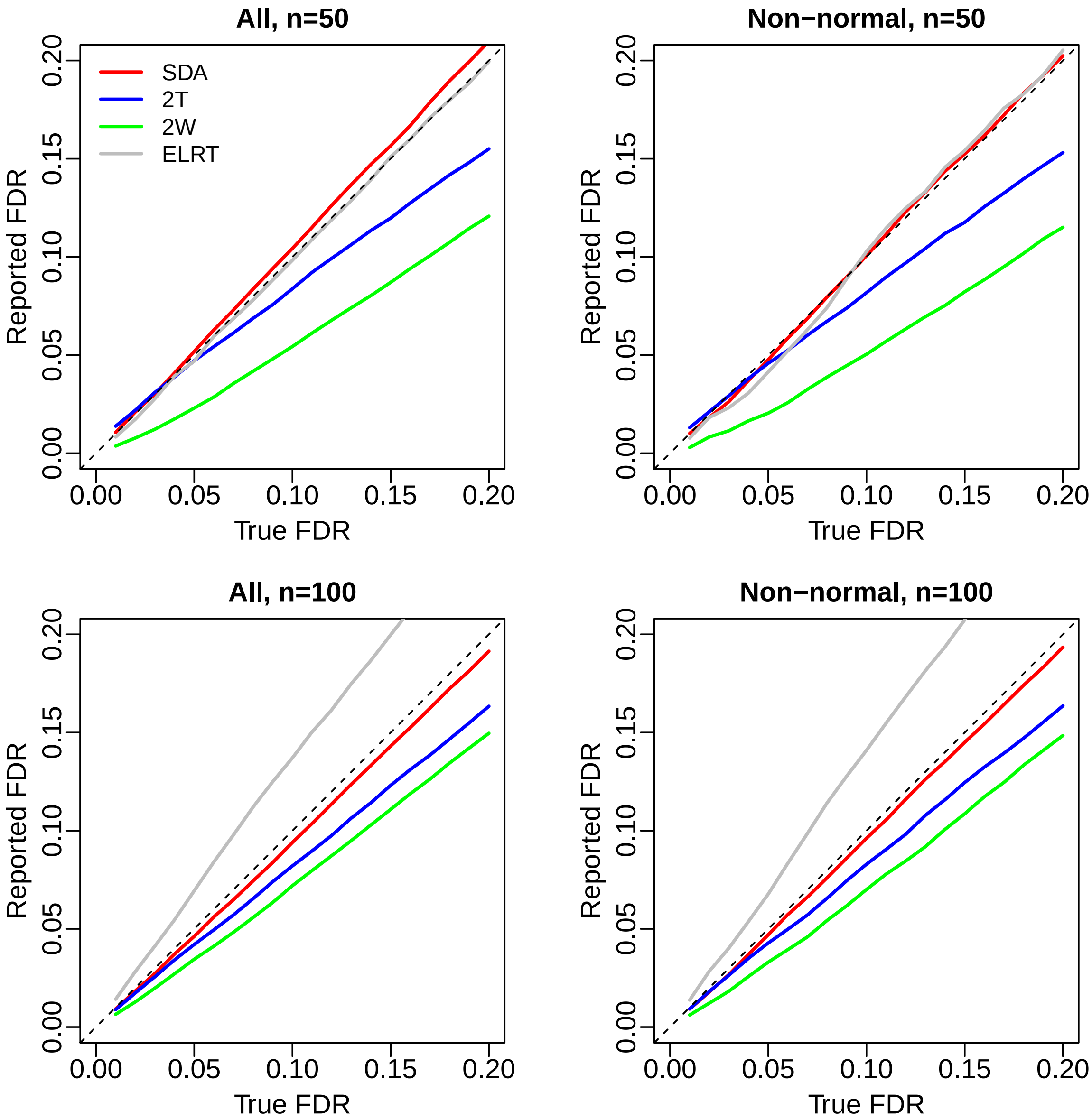

Figure S4: Comparison of false discovery rate (FDR) estimation when 20% of features were truly differentially abundant based on the first simulation scenario. Left panels: all features were considered; Right panels: only non-normal features (Shapiro-Wilk test p-value < 0.01 for at least one of the two groups) were considered. Results were averaged over 100 replicates.

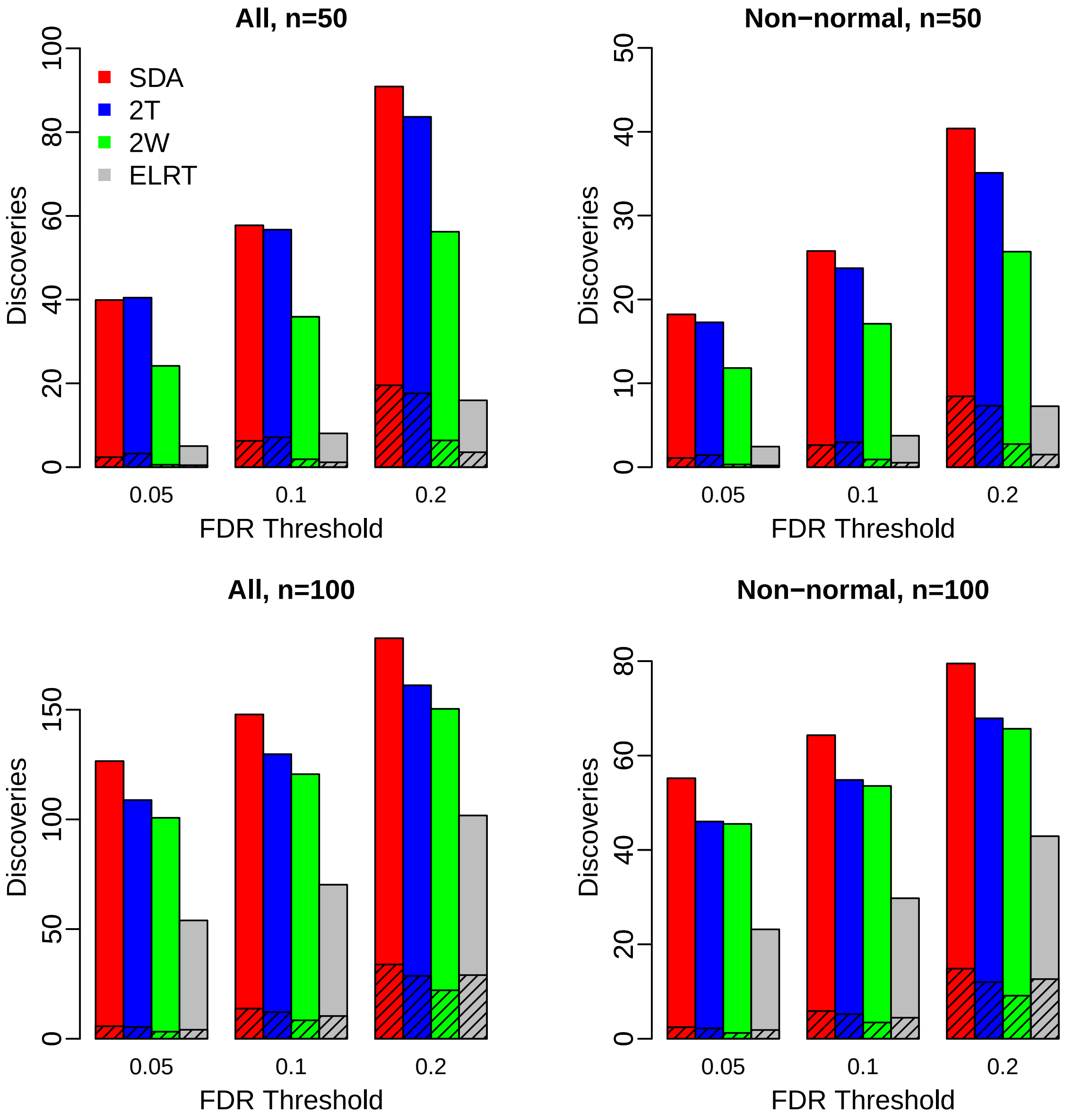

Figure S5: Comparison of the number of significant features for a FDR threshold of 0.05, 0.1, or 0.2 when 5% of features were truly differentially abundant based on the first simulation scenario. The unshaded bar indicates the number of true discoveries, and the shaded bar indicates the number of false discoveries. Results were averaged over 100 replicates. Left panels: all features were considered; Right panels: only non-normal features (Shapiro-Wilk test p-value < 0.01 for at least one of the two groups) were considered.

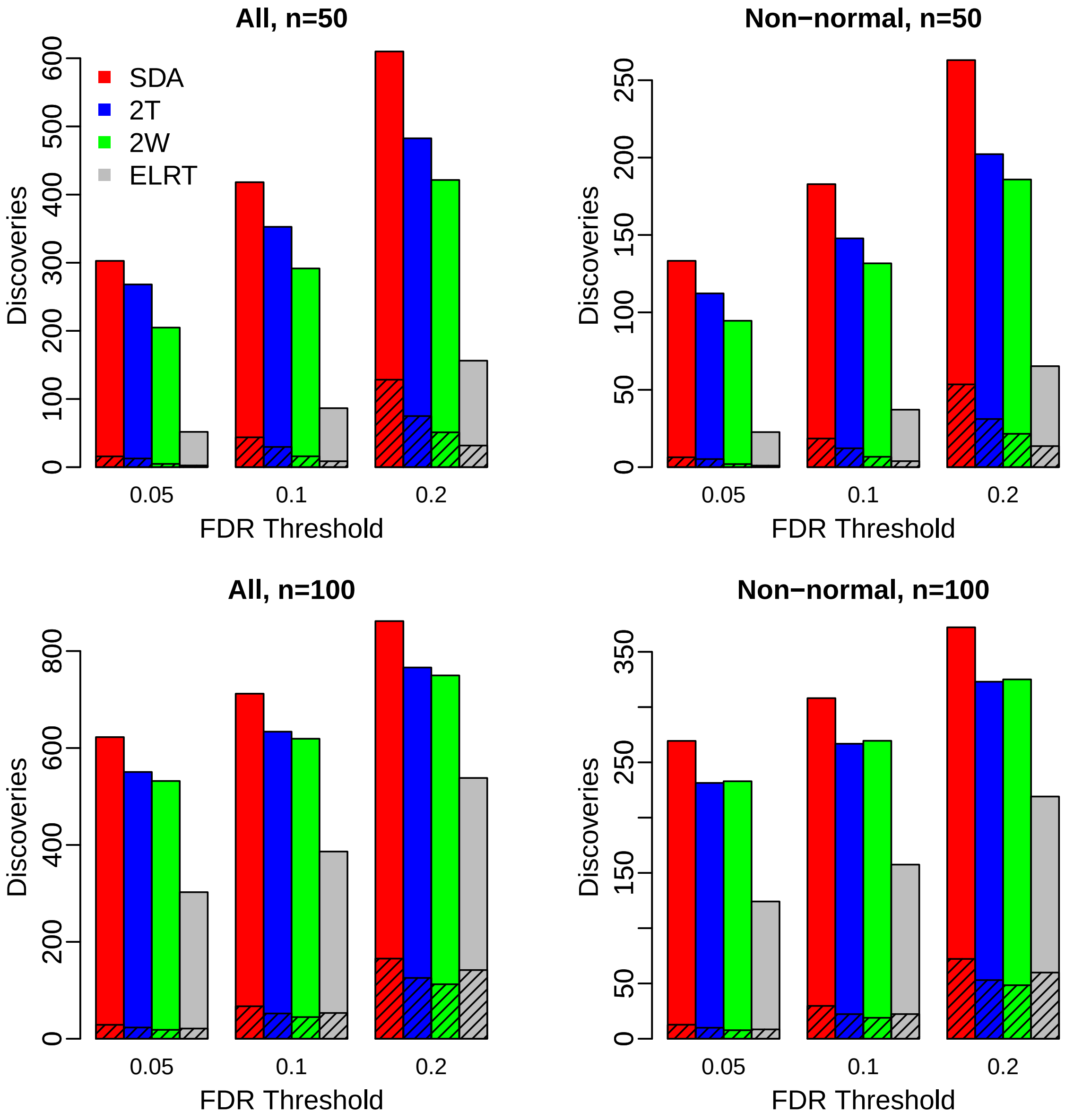

Figure S6: Comparison of the number of significant features for a FDR threshold of 0.05, 0.1, or 0.2 when 20% of features were truly differentially abundant based on the first simulation scenario. The unshaded bar indicates the number of true discoveries, and the shaded bar indicates the number of false discoveries. Results were averaged over 100 replicates. Left panels: all features were considered; Right panels: only non-normal features (Shapiro-Wilk test p-value < 0.01 for at least one of the two groups) were considered.

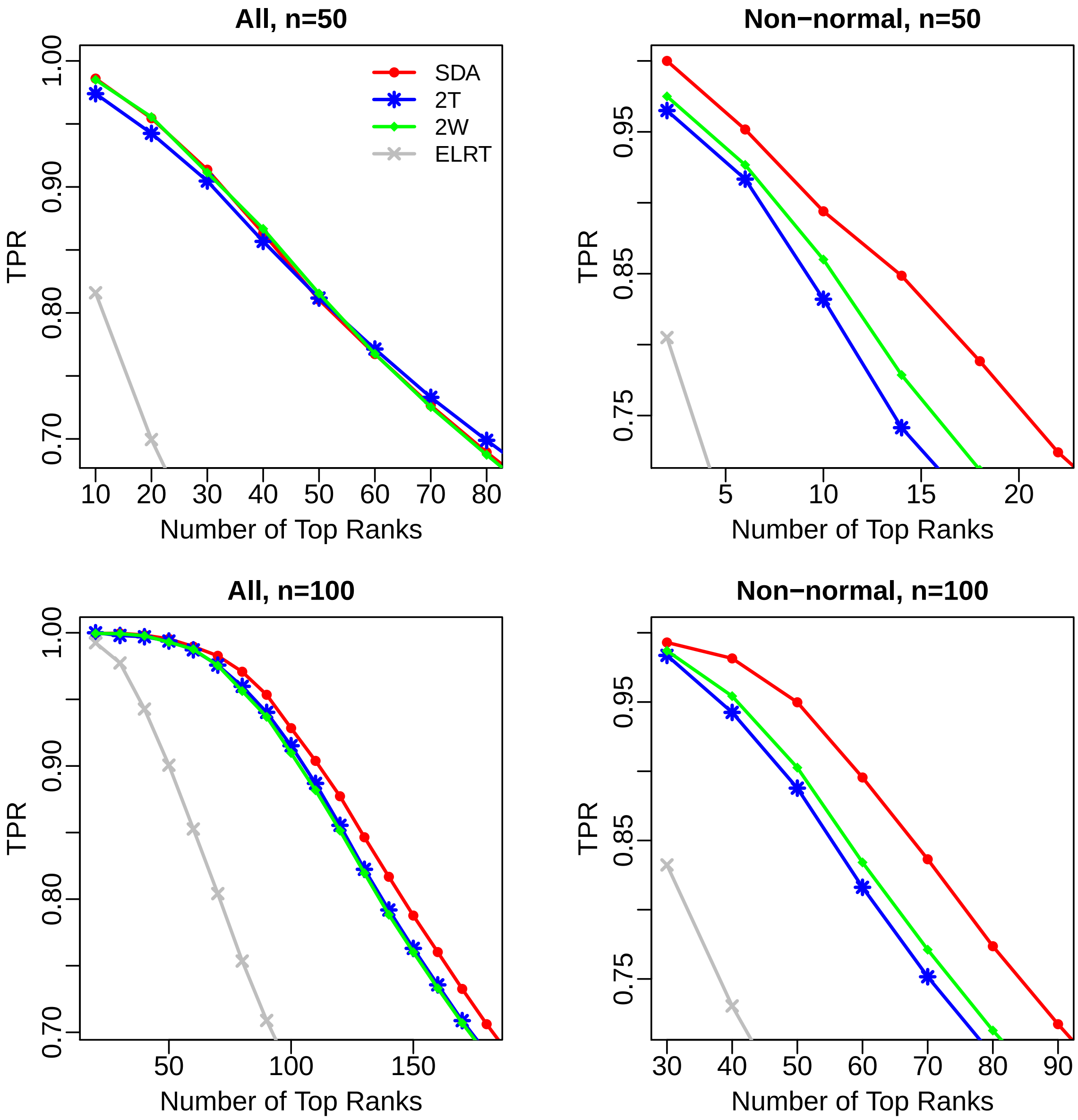

Figure S7: Comparison of the true positive rate (TPR) in top ranked features when 5% of features were truly differentially abundant based on the second simulation scenario. Left panels: all features were considered; Right panels: only non-normal features (Shapiro-Wilk test p-value < 0.01 for at least one of the two groups) were considered. The average TPR over 100 replicates was reported.

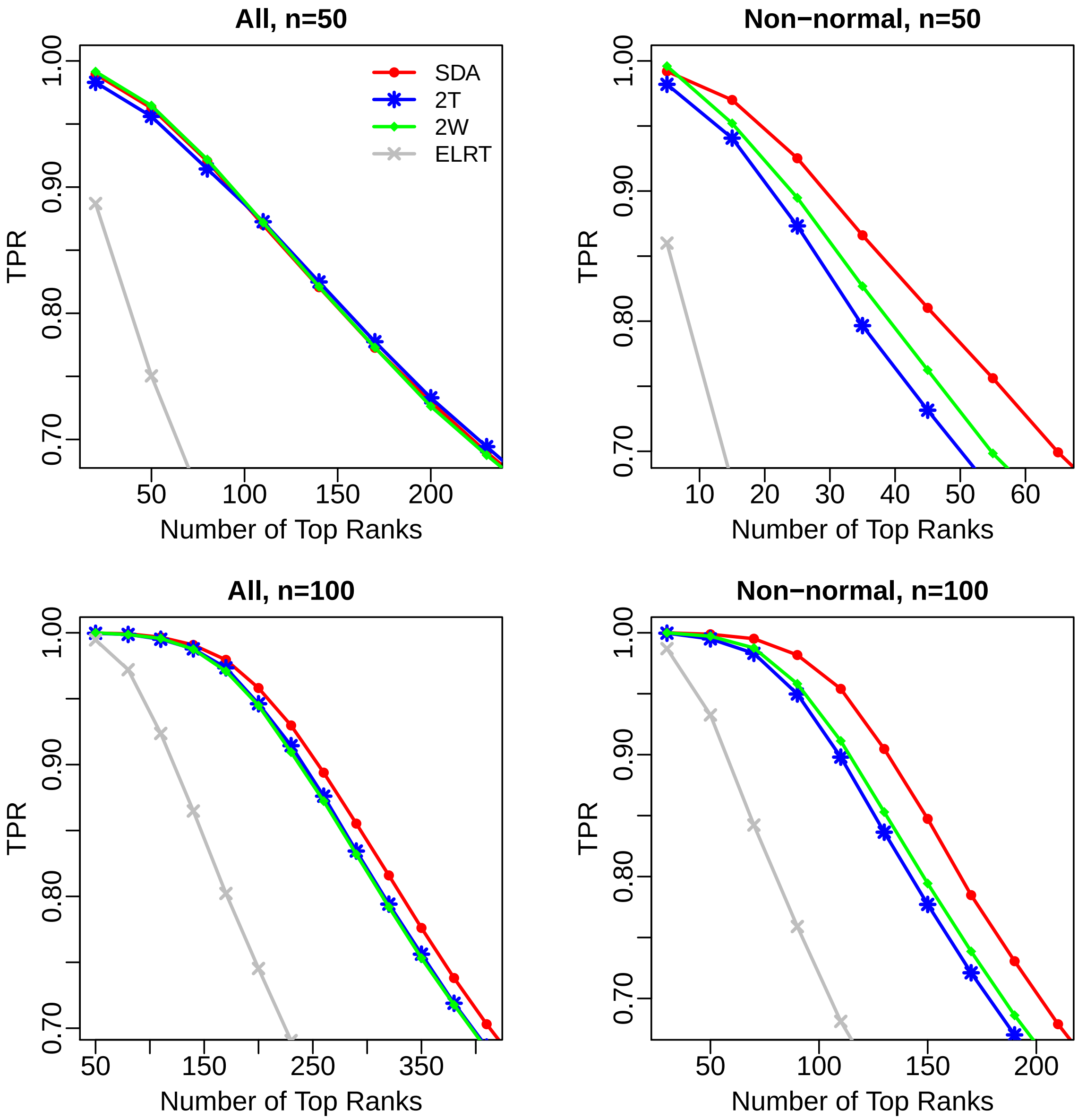

Figure S8: Comparison of the true positive rate (TPR) in top ranked features when 10% of features were truly differentially abundant based on the second simulation scenario Left panels: all features were considered; Right panels: only non-normal features (Shapiro-Wilk test p-value < 0.01 for at least one of the two groups) were considered. The average TPR over 100 replicates was reported.

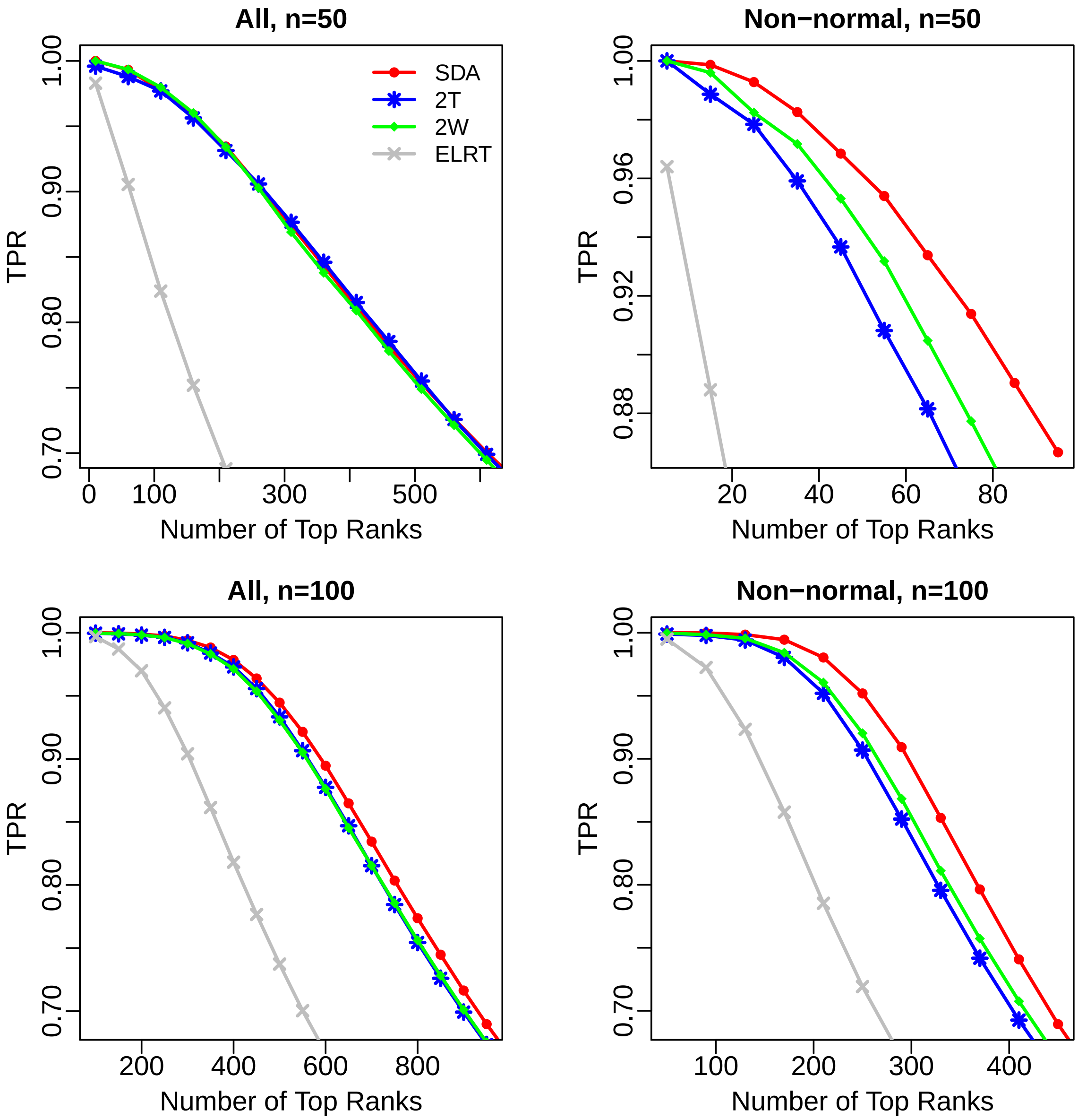

Figure S9: Comparison of the true positive rate (TPR) in top ranked features when 20% of features were truly differentially abundant based on the second simulation scenario. Left panels: all features were considered; Right panels: only non-normal features (Shapiro-Wilk test p-value < 0.01 for at least one of the two groups) were considered. The average TPR over 100 replicates was reported.

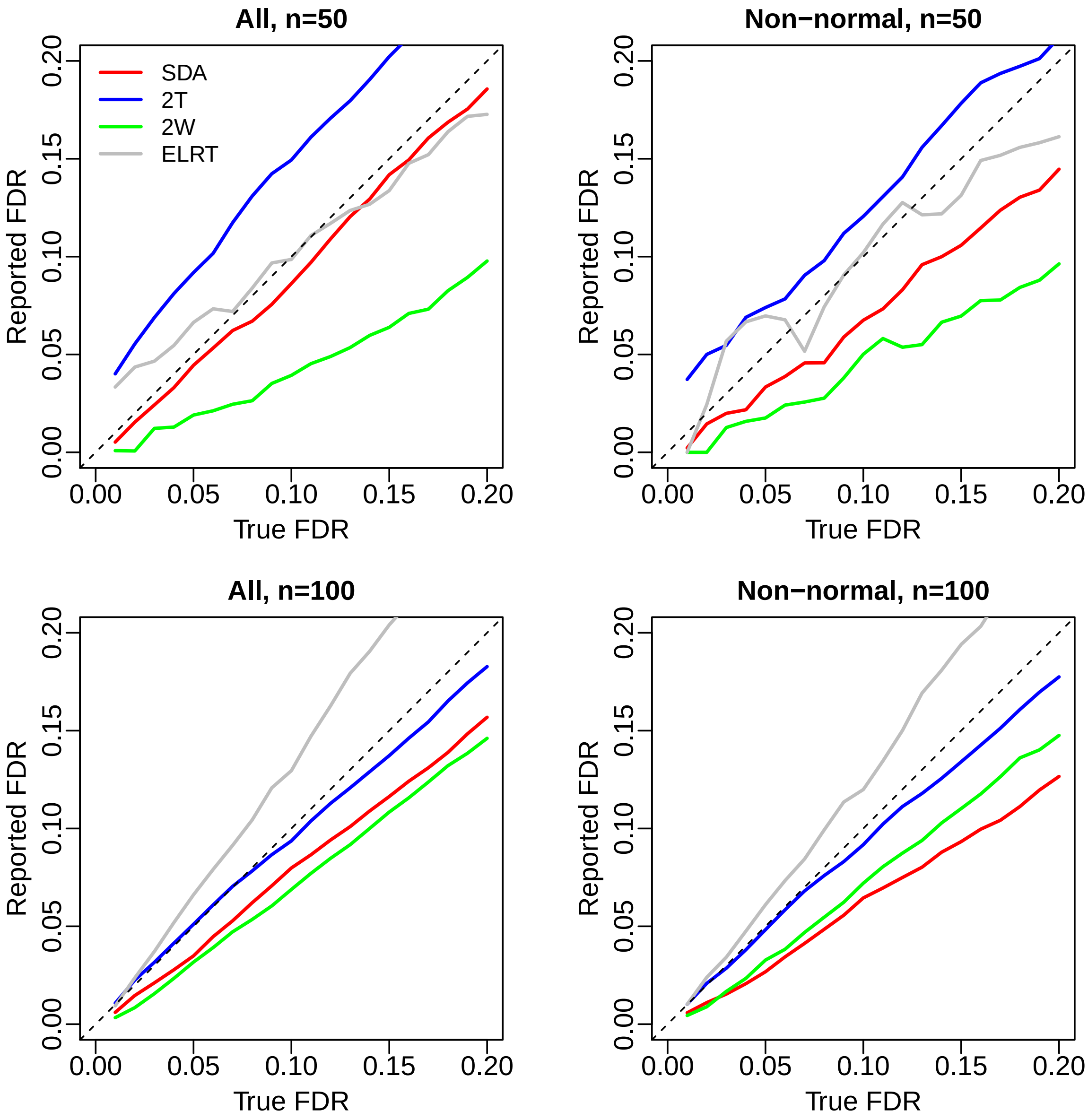

Figure S10: Comparison of false discovery rate (FDR) estimation when 5% of features were truly differentially abundant based on the second simulation scenario. Left panels: all features were considered; Right panels: only non-normal features (Shapiro-Wilk test p-value < 0.01 for at least one of the two groups) were considered. Results were averaged over 100 replicates.

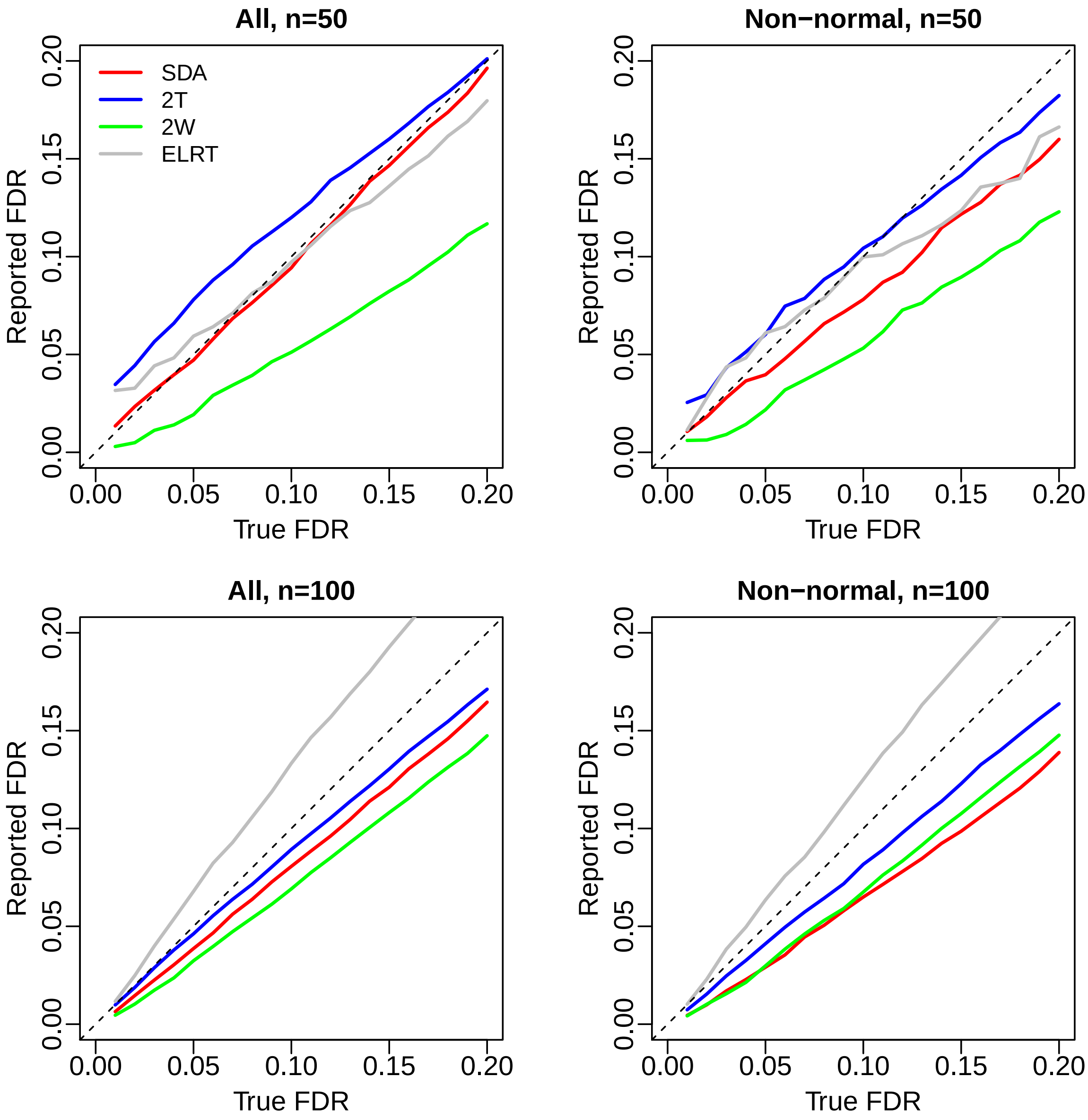

Figure S11: Comparison of false discovery rate (FDR) estimation when 10% of features were truly differentially abundant based on the second simulation scenario. Left panels: all features were considered; Right panels: only non-normal features (Shapiro-Wilk test p-value < 0.01 for at least one of the two groups) were considered. Results were averaged over 100 replicates.

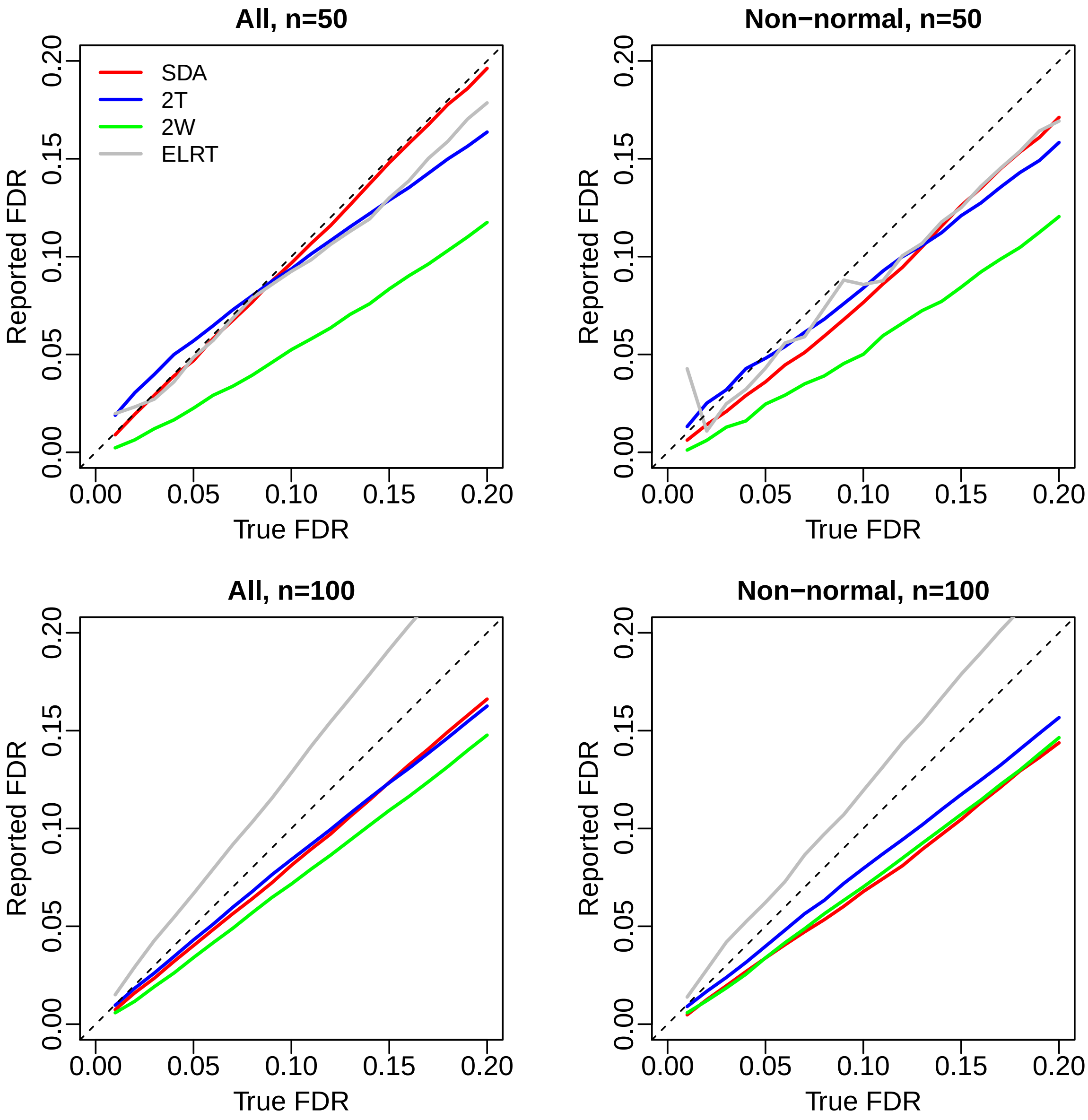

Figure S12: Comparison of false discovery rate (FDR) estimation when 20% of features were truly differentially abundant based on the second simulation scenario. Left panels: all features were considered; Right panels: only non-normal features (Shapiro-Wilk test p-value < 0.01 for at least one of the two groups) were considered. Results were averaged over 100 replicates.

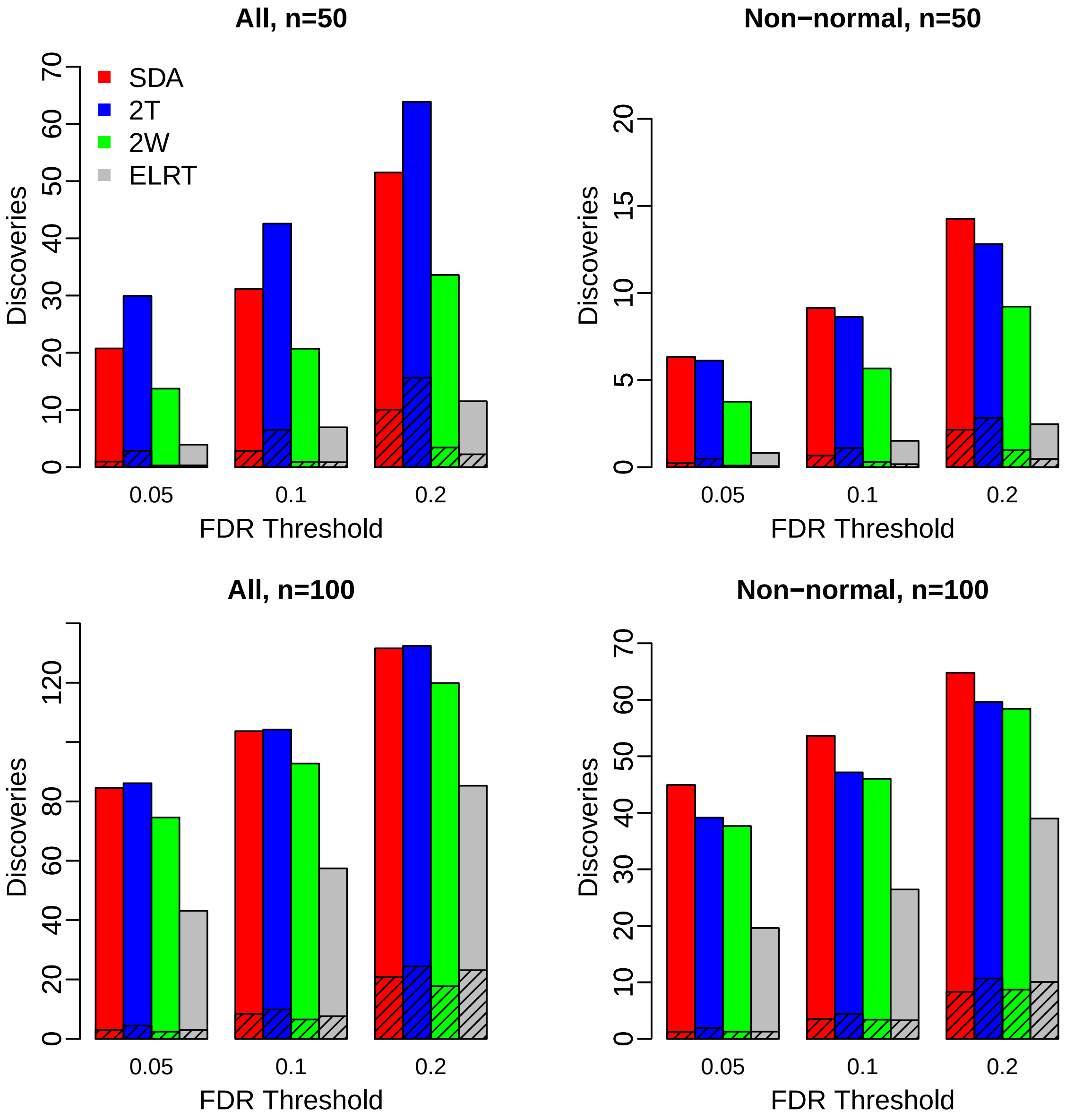

Figure S13: Comparison of the number of significant features for a FDR threshold of 0.05, 0.1, or 0.2 when 5% of features were truly differentially abundant based on the second simulation scenario. The unshaded bar indicates the number of true discoveries, and the shaded bar indicates the number of false discoveries. Results were averaged over 100 replicates. Left panels: all features were considered; Right panels: only non-normal features (Shapiro-Wilk test p-value < 0.01 for at least one of the two groups) were considered.

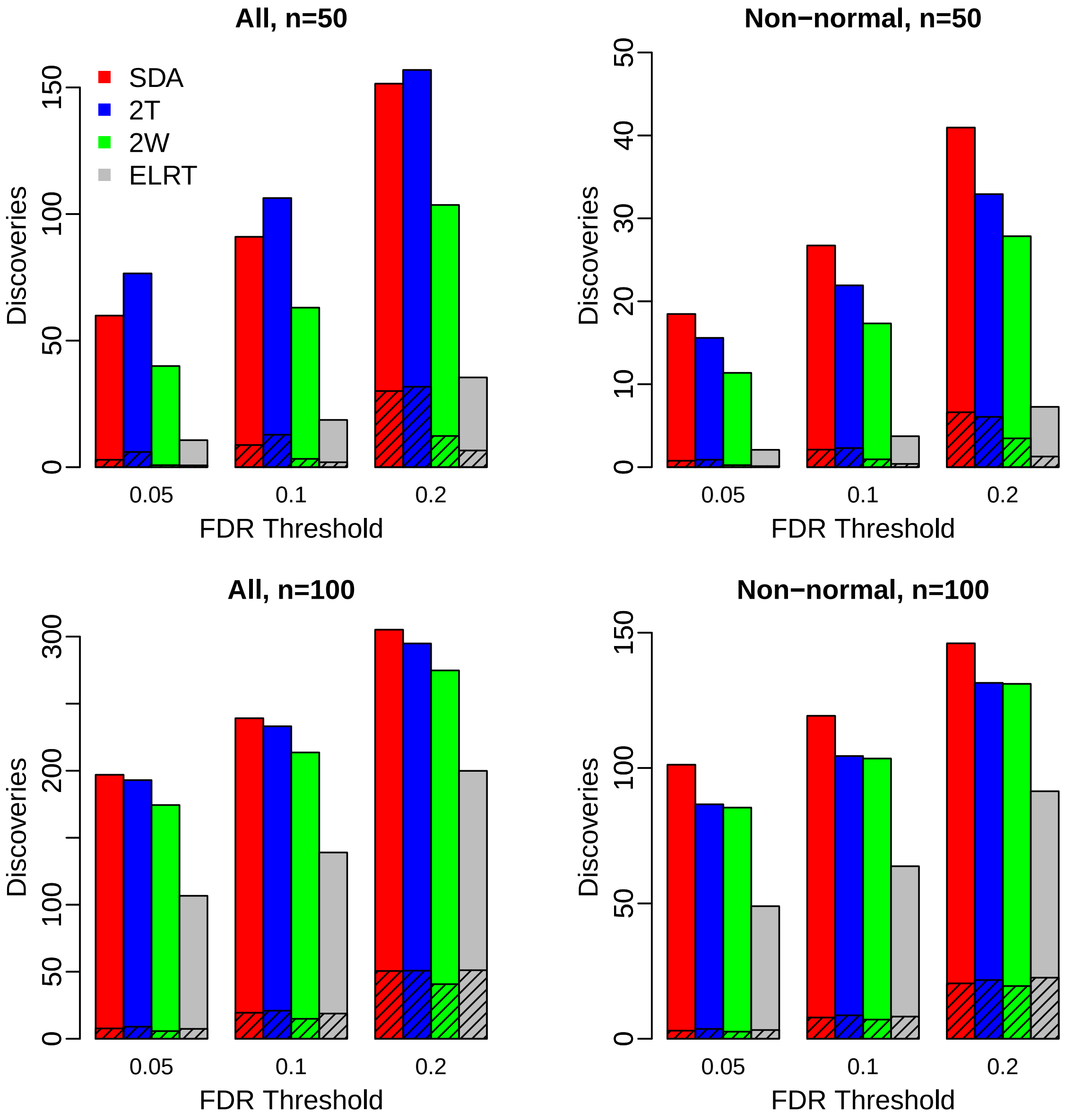

Figure S14: Comparison of the number of significant features for a FDR threshold of 0.05, 0.1, or 0.2 when 10% of features were truly differentially abundant based on the second simulation scenario. The unshaded bar indicates the number of true discoveries, and the shaded bar indicates the number of false discoveries. Results were averaged over 100 replicates. Left panels: all features were considered; Right panels: only non-normal features (Shapiro-Wilk test p-value < 0.01 for at least one of the two groups) were considered.

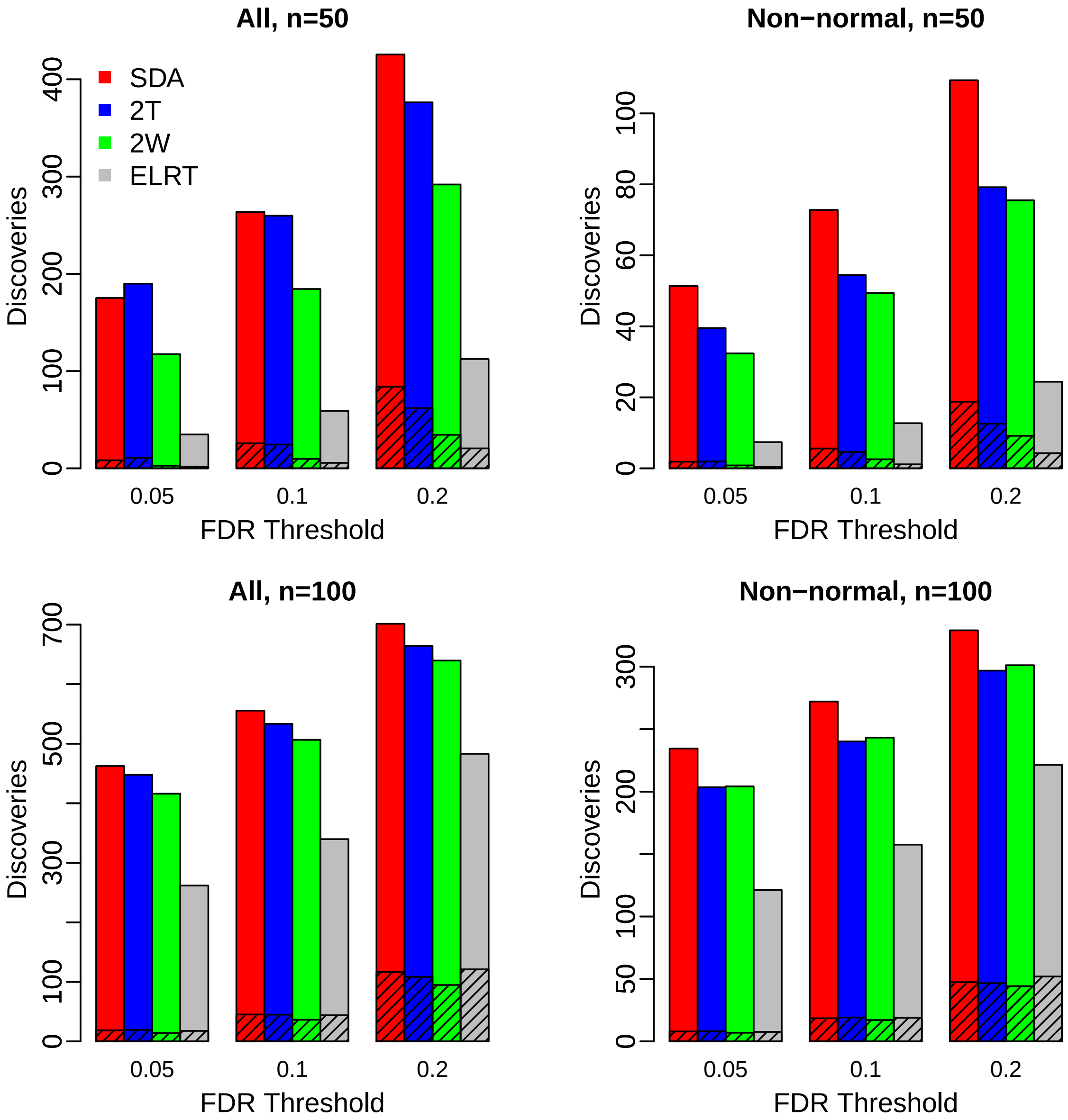

Figure S15: Comparison of the number of significant features for a FDR threshold of 0.05, 0.1, or 0.2 when 20% of features were truly differentially abundant based on the second simulation scenario. The unshaded bar indicates the number of true discoveries, and the shaded bar indicates the number of false discoveries. Results were averaged over 100 replicates. Left panels: all features were considered; Right panels: only non-normal features (Shapiro-Wilk test p-value < 0.01 for at least one of the two groups) were considered.

Table S2: Comparison of Type I error rates for all four methods. Data from simulation settings 1 and 2 were generated in the same way as the second simulation scenario in the main text, except that they were for a single normally (simulation setting 1) or non-normally (simulation settting 2) distributed feature. Data from simulation setting 3 were generated based on $LogNormal(0, 1)$. The significance level was set to 0.05.

Simulation setting	%zero	n	SDA	2T	2W	ELRT
1	33.7%	50	0.06	0.05	0.04	0.06
1	33.7%	100	0.06	0.05	0.05	0.07
2	74.7%	50	0.06	0.05	0.04	0.03
2	74.7%	100	0.06	0.05	0.04	0.05
3	50.0%	50	0.04	0.05	0.03	0.10
3	50.0%	100	0.05	0.05	0.05	0.08
