# Supplementary material for: SDA: a semi-parametric differential abundance analysis method for metabolomics and proteomics data
Source: BMC Bioinformatics. 2019 Oct 17;20:501. doi: 10.1186/s12859-019-3067-z (PMC6798423; doi:10.1186/s12859-019-3067-z)
Supplement: Supplementary file 2 — Additional file 2 Data acquisition procedure, data normalization and lipid assignment of differentially abundant features for the lung cancer exosomal lipid dataset. [file 12859_2019_3067_MOESM2_ESM.pdf]

Exosomes were purified from patient blood plasma by ultracentrifugation, and assessed for size distribution and particle numbers via the Nanosight300 instrument, as previously described [2]. Exosomal lipids were isolated by solvent extraction and characterized by ultra high resolution mass spectrometry using direct infusion ThermoFusion Orbitrap mass spectrometer interfaced to an Advion Nanomate as previously described in [2], except for 10 min total cycle time per sample for MS1 with 5 microscans per scan at a resolving power of 500,000 at 200 m/z and automatic gain control (AGC) target of 2e5. Monoisotopic precursor ions were isolated via quadrupole with 0.4 Da isolation window and higher energy collisional dissociation (HCD) with 25% collision energy was performed in positive mode. Fragmented ions were detected by Orbitrap with $R_{200m/z} = 500{,}000$. Lipids in each sample were assigned using the exact mass and fragment ions via in-house program PREMISE as described in [2]. Noise signals were removed by using intensity-independent noise filtering method for Fourier transform mass spectrometry [4] following ultra-high-resolution (R200m/z = 500,000)-based, modified resolution-dependent spectral binning [4]. Missing peaks (with intensities below a noise threshold) were assigned to zero, and known impurity peaks from blank runs were excluded form the master list. Ion intensities were normalized using the Nanosight300 data for each sample using the particle number density multiplied by the cube of the particle diameter, which represent the total amount of solute to be analyzed. After removing features with less than 10 non-zero values across samples, a total of 282 features were considered in differential abundance analysis. As shown in Table 2 of the main text, a total of 15 differentially abundant features were identified. Lipid assignments of those features are listed in the table below.

Table S1: Lipid Assignment

**MF**†	**adduct**	**exact mass**	**lipid ID**‡
C47H86O6	$[M+Na]^+$	769.631658	TAG (10:0_14:0_18:2)
			TAG (12:0_12:0_18:2)
C53H94O6	$[M+Na]^+$	849.694258	TAG (16:1_16:1_18:2)
			TAG (16:1_16:2_18:1)
			TAG (16:0_16:2_18:2)
			TAG (16:0_16:1_18:3)
			TAG (16:2_16:2_18:0)
C57H108O6*	$[M+Na]^+$	911.803808	TAG (10:0_14:0_18:2)
C59H104O6	$[M+Na]^+$	931.772508	TAG (16:0_18:2_22:3)
			TAG (16:0_18:1_22:4)
			TAG (16:0_20:1_20:4)
			TAG (16:0_20:2_20:3)
			TAG (18:0_18:1_20:4)
			TAG (18:0_18:2_20:3)
			TAG (18:1_18:2_20:2)
			TAG (18:1_18:1_20:3)
C54H100O6	$[M+Na]^+$	867.741208	TAG (16:0_17:0_18:2)

Continued on next page

**MF**†	**adduct**	**exact mass**	**lipid ID**‡
			TAG (16:1_17:0_18:1)
			TAG (16:2_17:0_18:0)
			TAG (18:1_15:0_18:1)
C49H92O6*	[M+Na]$^+$	799.678608	TAG (14:0_14:0_18:1)
			TAG (14:1_14:0_18:1)
			TAG (14:0_14:1_18:1)
			TAG (10:0_18:0_18:1)
			TAG (12:0_16:0_18:1)
			TAG (12:0_16:1_18:0)
C39H79N2O6P1*	[M+H]$^+$	703.574851	SM (18:1_16:0)
C40H80N1O8P1*	[M+H]$^+$	734.569432	PC (16:0_16:0)
C51H94O6*	[M+Na]$^+$	825.694258	TAG (14:0_16:1_18:1)
			TAG (14:1_16:1_18:0)
			TAG (14:1_16:0_18:1)
C52H98O6*	[M+Na]$^+$	841.725558	TAG (15:0_16:0_18:1)
C56H104O6*	[M+NH4]$^+$	890.817115	TAG (18:1_17:0_18:1)
			TAG (18:1_17:1_18:0)
			TAG (18:0_17:0_18:2)
C56H106O6	[M+NH4]$^+$	892.832765	TAG (18:1_17:0_18:0)
			TAG (18:0_17:1_18:0)
C59H106O6*	[M+Na]$^+$	933.788158	TAG (16:0_18:2_22:2)
			TAG (16:0_20:2_20:2)
			TAG (18:0_18:2_20:2)
			TAG (18:1_18:1_20:2)
			TAG (18:1_18:2_20:1)
C59H112O6	[M+NH4]$^+$	934.879715	TAG (16:0_18:0_22:1)
			TAG (16:0_18:1_22:0)
			TAG (16:1_18:0_22:0)
			TAG (18:0_18:0_20:1)
			TAG (18:0_18:1_20:0)
			TAG (18:1_18:0_20:0)
C56H102O6*	[M+Na]$^+$	893.756858	TAG (17:0_18:0_18:3)
			TAG (17:0_18:1_18:2)
			TAG (17:0_18:2_18:1)
			TAG (17:1_18:0_18:2)
			TAG (17:1_18:1_18:1)
			TAG (16:1_17:1_20:1)
			TAG (16:2_17:0_20:1)

† molecular formulae

‡ identified based on accurate mass and MS2 fragmentation patterns; SM, sphingomyelin; PC, phosphatidylcholine; TAG, triacylglyceride. Nomenclature is based on [1, 3].

References

[1] Eoin Fahy, Shankar Subramaniam, Robert C Murphy, Masahiro Nishijima, Christian RH Raetz, Takao Shimizu, Friedrich Spener, Gerrit van Meer, Michael JO Wakelam, and Edward A Dennis. Update of the lipid maps comprehensive classification system for lipids. *Journal of lipid research*, 50(Supplement):S9–S14, 2009.

[2] Teresa WM Fan, Xiaofei Zhang, Chi Wang, Ye Yang, Woo-Young Kang, Susanne Arnold, Richard M Higashi, Jinze Liu, and Andrew N Lane. Exosomal lipids for classifying early and late stage non-small cell lung cancer. *Analytica Chimica Acta*, 2018.

[3] Gerhard Liebisch, Juan Antonio Vizcaíno, Harald Köfeler, Martin Trötzmüller, William J Griffiths, Gerd Schmitz, Friedrich Spener, and Michael JO Wakelam. Shorthand notation for lipid structures derived from mass spectrometry. *Journal of lipid research*, 54(6):1523–1530, 2013.

[4] Kai Schuhmann, Henrik Thomas, Jacobo Miranda Ackerman, Konstantin O Nagornov, Yury O Tsybin, and Andrej Shevchenko. Intensity-independent noise filtering in ft ms and ft ms/ms spectra for shotgun lipidomics. *Analytical chemistry*, 89(13):7046–7052, 2017.
