# Supplementary material for: SDA: a semi-parametric differential abundance analysis method for metabolomics and proteomics data
Source: BMC Bioinformatics. 2019 Oct 17;20:501. doi: 10.1186/s12859-019-3067-z (PMC6798423; doi:10.1186/s12859-019-3067-z)
Supplement: Supplementary file 3 — Additional file 3 A proposition to show that for the case of two-group comparison in presence of detection limit, our test is also a valid test (in terms of preserving the type i error rate) to assess the null hypothesis that the distribution of underlying abundance level without censoring by the detection limit is the same between groups. [file 12859_2019_3067_MOESM3_ESM.pdf]

We consider the case of two-group comparison in presence of detection limit. Let Y_{ig}^* be the underlying abundance and D_{ig} be the detection limit for feature g in subject i, respectively. We assume $(Y_{ig}^*, D_{ig}), i = 1, \ldots, n$ are independent and identically distributed (i.i.d.). The observed abundance level is $Y_{ig} = Y_{ig}^* I\{Y_{ig}^* > D_{ig})$. Let $X_i = 0$ or 1 indicates the group assignment of subject i. For the two-group comparison situation, the two-part semiparametric model proposed in the main text is

$$\log(Y_{ig}) = \beta_g X_i + \epsilon_{ig} \text{ for } Y_{ig} > 0 \text{ and } \log\left(\frac{\pi_{ig}}{1-\pi_{ig}}\right) = \gamma_{0g} + \gamma_g X_i \text{ for } Y_{ig} = 0,$$

where $\epsilon_{ig}, i = 1, \ldots, N$ are i.i.d. random errors with an unspecified distribution and are independent with X_i.

Under the null hypothesis $H_0 : \beta_g = 0$ and $\gamma_g = 0$, we have

$$\log(Y_{ig}) = \epsilon_{ig} \text{ for } Y_{ig} > 0 \text{ and } \log\left(\frac{\pi_{ig}}{1-\pi_{ig}}\right) = \gamma_{0g} \text{ for } Y_{ig} = 0. \tag{S1}$$

The above model is for observed abundance Y_{ig} and the likelihood ratio test LRT_g we construct in the main text is to compare the distribution of Y_{ig} between groups. Note that since the distribution of ϵ_{ig} is completely unspecified, model (S1) holds as long as Y_{ig}'s are i.i.d. and independent of X_i. Because of this nice property, LRT_g can also be used to compare the distribution of Y_{ig}^* between groups, as stated in the following proposition.

Proposition S1. For two-group comparison, assuming that the detection limit D_{ig} is independent of group X_i, the likelihood ratio test LRT_g proposed in the main text is also a valid test for

$$\begin{aligned} H_0^* &: \text{ the distribution of } Y_{ig}^* \text{ is the same between groups} \\ \text{v.s. } H_1^* &: \text{ the distribution of } Y_{ig}^* \text{ is different between groups} \end{aligned}$$

in the sense that the type I error rate is preserved.

Proof: The critical region of the likelihood ratio test is $\{LRT_g > \chi^2_{2,1-\alpha}\}$, where $P(LRT_g > \chi^2_{2,1-\alpha} | H_0) = \alpha$, where $\chi^2_{2,1-\alpha}$ is the $1-\alpha$ quantile of a chi-square distribution with 2 degrees of freedom and α is the type I error rate. We next show that hypothesis H_0^* implies H_0 so that LRT_g is also a valid test for H_0^*. Specifically, H_0^* implies that Y_{ig}^* is independent with X_i. Because $Y_{ig} = Y_{ig}^* I\{Y_{ig}^* > D_{ig})$ and D_{ig} is independent with X_i, Y_{ig} is also independent with X_i. Thus, the distribution of Y_{ig} satisfies equation (S1) and H_0 holds. This implies that $P(LRT_g > \chi^2_{2,1-\alpha} | H_0^*) = P(LRT_g > \chi^2_{2,1-\alpha} | H_0) = \alpha$. Therefore, LRT_g is a valid test for H_0^*.

Remark 1. Our test LRT_g preserves the type I error rate regardless of the distribution of the underlying abundance level Y_{ig}^*.

Remark 2. If our test is rejected, it indicates there is difference in the distribution of Y_{ig}^* between groups. But the parameter estimate, $\hat{\beta}_g$, obtained from our model quantifies the log fold change of observed abundance level between groups rather than that of underlying abundance level.
